# Gene expression of transporters and phase I/II metabolic enzymes in murine small intestine during fasting

**DOI:** 10.1186/1471-2164-8-267

**Published:** 2007-08-07

**Authors:** Heleen M van den Bosch, Meike Bünger, Philip J de Groot, Jolanda van der Meijde, Guido JEJ Hooiveld, Michael Müller

**Affiliations:** 1 Nutrition, Metabolism and Genomics group, Division of Human Nutrition, Wageningen, University, Bomenweg 2, 6703 HD Wageningen, The Netherlands; 2 Nutrigenomics Consortium, TI Food and Nutrition, Wageningen, The Netherlands

## Abstract

**Background:**

Fasting has dramatic effects on small intestinal transport function. However, little is known on expression of intestinal transport and phase I/II metabolism genes during fasting and the role the fatty acid-activated transcription factor PPARα may play herein. We therefore investigated the effects of fasting on expression of these genes using Affymetrix GeneChip MOE430A arrays and quantitative RT-PCR.

**Results:**

After 24 hours of fasting, expression levels of 33 of the 253 analyzed transporter and phase I/II metabolism genes were changed. Upregulated genes were involved in transport of energy-yielding molecules in processes such as glycogenolysis (*G6pt1*) and mitochondrial and peroxisomal oxidation of fatty acids (*Cact*, *Mrs3/4*, *Fatp2*, *Cyp4a10*, *Cyp4b1*). Other induced genes were responsible for the inactivation of the neurotransmitter serotonin (*Sert*, *Sult1d1*, *Dtd*, *Papst2*), formation of eicosanoids (*Cyp2j6*, *Cyp4a10*, *Cyp4b1*), or for secretion of cholesterol (*Abca1 *and *Abcg8*). Cyp3a11, typically known because of its drug metabolizing capacity, was also increased. Fasting had no pronounced effect on expression of phase II metabolic enzymes, except for glutathione *S*-transferases which were down-regulated. Time course studies revealed that some genes were acutely regulated, whereas expression of other genes was only affected after prolonged fasting. Finally, we identified 8 genes that were PPARα-dependently upregulated upon fasting.

**Conclusion:**

We have characterized the response to fasting on expression of transporters and phase I/II metabolic enzymes in murine small intestine. Differentially expressed genes are involved in a variety of processes, which functionally can be summarized as a) increased oxidation of fat and xenobiotics, b) increased cholesterol secretion, c) increased susceptibility to electrophilic stressors, and d) reduced intestinal motility. This knowledge increases our understanding of gut physiology, and may be of relevance for e.g. pre-surgery regimen of patients.

## Background

Fasting, the act of willingly abstaining from food, is a frequently occurring natural status in humans. Fasting is a popular strategy to manage overweight or obesity, it is a traditional habit in certain religions or societies, and it is an accepted pre-surgical procedure. During fasting whole-body fuel utilization gradually shifts from carbohydrates and fat in the fed state to proteins and fat after a day of fasting [1]. The nuclear receptor peroxisome proliferator-activated receptor *alpha *(PPARα) plays an important role in the control of the hepatic metabolic response [2]. During fasting, free fatty acid levels in plasma are elevated and can activate PPARα, which regulates a large array of hepatic genes including those involved in fatty acid catabolism.

The small intestine is the primary organ for digestion and selective absorption of nutrients and other food constituents. Absorption of these molecules across the intestinal epithelium occurs mainly by multiple transmembrane transporters [3-6] that principally belong to two superfamilies, namely the solute carrier (SLC) and the ATP Binding Cassette (ABC) superfamily of transporters [5,7]. SLC transporters located at the apical membrane of the enterocyte are responsible for the selective uptake of macronutrients, such as di- and tripeptides, hexoses and fatty acids [8]. In contrast, ABC transporters are efflux transporters responsible for the active removal of substances, including nutrients such as cholesterol, limiting their intracellular concentrations. Besides their presence in plasma membranes, SLC and ABC transporters are also located in intracellular organelles, such as mitochondria or peroxisomes, in which they are responsible for uptake or secretion of metabolites.

In addition, it has become clear that the intestinal epithelium is an important metabolic site, to a great extend responsible for the first-pass metabolism of nutrients and xenobiotics [9,10]. Numerous metabolic reactions occur in enterocytes, including those typically referred to as phase I and phase II metabolism. Phase I metabolism commonly refers to oxidative, peroxidative, and reductive metabolism of endogenous compounds and drugs, mediated by cytochrome P450 isoenzymes (CypP450s) [11]. Phase II metabolism often succeed phase I metabolism and is mediated by several enzymatic systems. In general, phase II metabolism yields conjugated metabolites, increasing the water solubility of lipophilic compounds. The most important phase II enzymes are sulfotransferases (Sults) [12,13], UDP-glucuronosyltransferases (Ugts) [14], glutathione S-transferases (Gsts) [15,16], N-acetyltransferases (Nats) [17], and epoxide hydrolases (Ephs) [18]. Several ABC transporters can secrete metabolites resulting from phase I and phase II enzymatic transformations [19].

Previous studies showed that fasting has a dramatic effect on small intestinal transport function [20]. However, little is known on the expression of transport and phase I/II metabolism genes in small intestine during fasting and the role of PPARα therein. We therefore set out to investigate the effects of fasting on expression of these genes using microarrays and quantitative RT-PCR (qRT-PCR). We conclude that the absorptive as well as the detoxification capacity of the small intestine is altered during fasting, and that PPARα mediates a part of the adaptive response to fasting.

## Results

### Effect of 24 hours of fasting on expression of transporter and phase I/II metabolism genes

The Affymetrix GeneChip Mouse Genome 430A array comprises 22,690 probesets, representing 12,453 unique genes. Annotation information from Affymetrix was queried to compile a list of transporter and phase I/II metabolism genes present on the array (for details, see Methods). This list consisted of 665 probesets, encoding for 436 unique genes, and was used in the remainder of our analyses (Table 1). Under basal, chow-fed conditions, our filtering protocol identified 5,993 significantly expressed genes in the small intestine (i.e. having an absolute expression signal >20), of which 253 were transporters and phase I/II metabolism genes. After 24 hours of fasting, 713 genes, including 33 transporter and phase I/II metabolism genes, were differentially expressed (fold change >1.3, p-value < 0.01), corresponding to 13% of the expressed transporter and phase I/II metabolism genes. For selected genes additional qRT-PCR analyses were performed, which confirmed the array results (Table 2).

**Table 1:** Numbers of expressed and regulated genes in small intestine analyzed on Affymetrix GeneChip MOE430A arrays.

	**All genes**	**Transporters + phase I/II metabolism genes**
Number of probe sets on MOE430A array	22690	665
Number of unique genes on MOE430A array	12453	436
Expressed genes on MOE430A array	5993	253
Regulated after 24 hours of fasting	713	33
Regulated genes, as % of expressed genes	11.8	13.0

Analysis of all genes on the MOE430A array compared with transporters and phase I/II metabolism genes.

**Table 2:** Confirmation of microarray results.

**Gene symbol**	**Affy probe set ID**	**FC microarray**	**P-value microarray**	**FC qRT-PCR**	**P-value qRT-PCR**	**Gene name**
*Cyp4a10*	1424853_s_at	3.6	0.0019	2.5 ± 0.34	0.0172	cytochrome P450, family 4, subfamily a, polypeptide 10
*Cyp2j6*	1417952_at	2.3	0.0000	1.7 ± 0.32	0.0435	cytochrome P450, family 2, subfamily j, polypeptide 6
*Abca1*	1421840_at	2.3	0.0005	2.4 ± 0.26	0.0330	ATP-binding cassette, sub-family A (ABC1), member 1
*G6pt1 (Slc37a4)*	1417042_at	2.3	0.0010	1.9 ± 0.20	0.0086	solute carrier family 37 (glycerol-6-phosphate transporter), member 4
*Znt2 (Slc30a2)*	1427339_at	1.9	0.0002	1.6 ± 0.24	0.0383	solute carrier family 30 (zinc transporter), member 2
*Abcg8*	1420656_at	1.8	0.0003	1.7 ± 0.29	0.0492	ATP-binding cassette, sub-family G (WHITE), member 8
*Sult1d1*	1418138_at	1.7	0.0017	1.8 ± 0.33	0.0310	sulfotransferase family 1D, member 1
*Fatp2 (Slc27a2)*	1416316_at	1.6	0.0003	2.0 ± 0.24	0.0283	solute carrier family 27 (fatty acid transporter), member 2
*Nadc1 (Slc13a2)*	1418857_at	1.6	0.0011	1.6 ± 0.13	0.0247	solute carrier family 13 (sodium-dependent dicarboxylate transporter), member 2
*Slc25a36*	1419656_at	1.5	0.0007	2.0 ± 0.19	0.0114	solute carrier family 25, member 36
*Chst4*	1453393_a_at	1.4	0.0008	1.6 ± 0.13	0.0142	carbohydrate (chondroitin 6/keratan) sulfotransferase 4
*Dtd (Slc26a2)*	1421145_at	1.4	0.0028	1.5 ± 0.06	0.0017	solute carrier family 26 (sulfate transporter), member 2
*Mrs3/4 (Slc25a28)*	1424776_a_at	1.4	0.0018	1.5 ± 0.08	0.0141	solute carrier family 25, member 28
*Mgst1*	1415897_a_at	1.3	0.0014	2.0 ± 0.21	0.0078	microsomal glutathione S-transferase 1
*Cyp3a11*	1416809_at	1.3	0.0057	1.9 ± 0.22	0.0149	cytochrome P450, family 3, subfamily a, polypeptide 11
*Mct4 (Slc16a3)*	1449005_at	-1.6	0.0058	-1.6 ± 0.00	0.0329	solute carrier family 16 (monocarboxylic acid transporters), member 3
*Zip4 (Slc39a4)*	1451139_at	-2.4	0.0003	-2.2 ± 0.18	0.0472	solute carrier family 39 (zinc transporter), member 4
*Gstm3*	1427473_at	-2.6	0.0027	-2.2 ± 0.08	0.0338	glutathione S-transferase, mu 3

Microarray results were confirmed with qRT-PCR. FC = Fold change, qRT-PCR control samples have been set arbitrarily to 1, qRT-PCR data are means ± standard error (n = 3).

#### Solute carrier transporters

We studied 243 SLC transporters, which amounts to 68% of the total number transporters of this superfamily currently known (see additional file 1). After 24 hours of fasting, 16 SLC transporters were differentially expressed (Table 3), which corresponded to 15% of the expressed SLC transporter genes in the small intestine. With respect to apical transporters, expression of the short-chain fatty acid transporter *Smct1 *(*Slc5a8*) [21], the carboxylate transporter *Nadc1 *(*Slc13a2*) [22], the prostaglandin transporter *Pgt *(*Slco2a1*) [23], and the sulphate transporter *Dtd *(*Slc26a2*) [24] was increased, whereas the expression level of the zinc transporter *Zip4 *(*Slc39a4*) [25] was suppressed. The basolaterally located monocarboxylate transporter *Mct4 *(*Slc16a3*) [26] and iron transporter *Ireg1 *(*Slc40a1*) [27] were downregulated, and the basolateral neurotransmitter serotonin transporter *Sert *(*Slc6a4*/*5-HTT*) [28], was upregulated. Seven intracellular SLC transporters were induced (Table 3); *Znt2 *(*Slc30a2*), *G6pt1 *(*Slc37a4*), *Fatp2 *(*Slc27a2*), *Papst2 *(*Slc35b3*), *Cact *(*Slc25a20*), *Mrs3/4 *(*Slc25a28*), and *Slc25a36*. *Znt2 *[29] is responsible for storage of zinc in sub-apically-located vesicles. *G6pt1 *[30] and *Fatp2 *[31] are localized at the endoplasmic reticulum, transporting glucose-6 phosphate and fatty acyl-CoA esters, respectively. The sulphate donor 3'-phosphoadenosine 5'-phosphosulfate (PAPS) transporter *Papst2 *[32] is localized in the Golgi. Finally, *Cact*, *Mrs3/4 *and *Slc25a36 *are all three present in mitochondria shuttling metabolites across the inner mitochondrial membrane [33]. *Cact *and *Mrs3/4 *transport fatty acyl carnitines for fatty acid oxidation and iron, respectively. The function of *Slc25a36 *is currently not known. Finally, *Fuct1 *(*Slc35c1*) [34] an additional member of the Slc35 family, transporting nucleotide sugars, was downregulated.

**Table 3:** Differential expressed SLC transporters in the small intestine after 24 h fasting.

**Gene symbol**	**Affy probe set ID**	**A value**	**SD WT 0 hr**	**SD WT 24 hr**	**Fold change**	**P-value**	**Localization**	**Gene name**
*G6pt1 (Slc37a4)*	1417042_at	7.7	0.44	0.37	2.3	0.0010	Endoplasmatic reticulum	Solute carrier family 37 (glycerol-6-phosphate transporter), member 4
*Znt2 (Slc30a2)*	1427339_at	5.6	0.09	0.30	1.9	0.0002	Vesicles	Solute carrier family 30 (zinc transporter), member 2
*Slc25a36*	1419656_at	6.7	0.09	0.22	1.7	0.0002	Mitochondria	Solute carrier family 25, member 36
*Fatp2(Slc27a2)*	1416316_at	10.4	0.19	0.11	1.6	0.0003	Peroxisomes and ER	Solute carrier family 27 (fatty acid transporter), member 2
*Nadc1 (Slc13a2)*	1418857_at	10.2	0.23	0.15	1.6	0.0011	Apical	Solute carrier family 13 (sodium-dependent dicarboxylate transporter), member 2
*Slc25a36*	1419657_a_at	9.4	0.16	0.14	1.5	0.0007	Mitochondria	Solute carrier family 25, member 36
*Papst2 (Slc35b3)*	1448937_at	7.6	0.04	0.18	1.5	0.0005	Golgi	Solute carrier family 35, member B3
*Smct1 (Slc5a8)*	1425606_at	6.5	0.14	0.12	1.5	0.0009	Apical	Solute carrier family 5 (iodide transporter), member 8
*Cact (Slc25a20)*	1423108_at	9.2	0.04	0.07	1.4	0.0002	Mitochondria	Solute carrier family 25 (mitochondrial carnitine/acylcarnitine translocase), member 20
*Cact (Slc25a20)*	1423109_s_at	7.9	0.10	0.19	1.4	0.0019	Mitochondria	Solute carrier family 25 (mitochondrial carnitine/acylcarnitine translocase), member 20
*Sert (Slc6a4)*	1417150_at	8.5	0.18	0.07	1.4	0.0014	Basolateral	Solute carrier family 6 (neurotransmitter transporter, serotonin), member 4
*Mrs3/4 (Slc25a28)*	1424776_a_at	7.0	0.12	0.17	1.4	0.0018	Mitochondria	Solute carrier family 25, member 28
*Dtd (Slc26a2)*	1421145_at	5.6	0.06	0.22	1.4	0.0028	Apical	Solute carrier family 26 (sulfate transporter), member 2
*Oatp2a1 (Slco2a1)*	1420913_at	8.3	0.06	0.17	1.4	0.0023	Apical	Solute carrier organic anion transporter family, member 2a1
*Fuct1 (Slc35c1)*	1452139_at	7.3	0.16	0.36	-1.4	0.0052	Golgi	Solute carrier family 35, member C1
*Ireg1 (Slc40a1)*	1417061_at	8.1	0.38	0.35	-1.6	0.0099	Basolateral	Solute carrier family 40 (iron-regulated transporter), member 1
*Mct4 (Slc16a3)*	1449005_at	4.2	0.44	0.21	-1.6	0.0058	Basolateral	Solute carrier family 16 (monocarboxylic acid transporters), member 3
*Ireg1 (Slc40a1)*	1448566_at	7.8	0.48	0.50	-1.7	0.0084	Basolateral	Solute carrier family 40 (iron-regulated transporter), member 1
*Zip4 (Slc39a4)*	1451139_at	9.0	0.04	0.48	-2.0	0.0003	Apical	Solute carrier family 39 (zinc transporter), member 4

A = the average log2 transformed expression value of normal fed and 24 hours fasted mice (n = 3), SD = standard deviation, WT = wild-type mice. In addition to the fold change, the intracellular localization of the corresponding protein is given.

#### Phase I and II metabolic genes

Next we evaluated the effects of fasting on expression of phase I/II metabolism genes (see additional file 2). Results are presented in Table 4. We studied 61 CypP450s, which corresponds to 66% of the currently known mouse CypP450s. Five CypP450s were differentially expressed, corresponding to 29% of the expressed CypP450s in small intestine. *Cyp2j6 *was upregulated. Members of the Cyp2 family are well known to be responsible for the NADPH-dependent oxidation of steroids and fatty acid as well as drugs [11]. In addition, expression of *Cyp3a11*, typically known because of its drug-metabolizing capacity [11], was increased. Finally, *Cyp4a10 *and *Cyp4b1*, involved in peroxisomal oxidation of fatty acids, and *Cyp27a1*, involved in conversion of cholesterol into 27-hydroxycholesterol, were all induced upon fasting [11,35,36]. With respect to phase II metabolism, expression of in total 89 enzymes was analyzed (see additional file 2). Except for Gsts, fasting had no denoting effect on expression of phase II metabolism genes (Table 4), since only 3 non-Gsts were changed. Apart from *Gstm1*, all changed Gsts were downregulated. The aldo-keto reductase *Akr1b7 *[37], involved in detoxification of lipid peroxides, and two sulfotransferases, *Sult1d1 *and *Chst4*, were increased.

**Table 4:** Differential expressed detoxification genes in the small intestine after 24 h fasting.

**Gene symbol**	**Affy probe set ID**	**A value**	**SD WT 0 hr**	**SD WT 24 hr**	**Fold change**	**P-value**	**Gene name**
**CYPP450s**							
*Cyp4a10*	1424853_s_at	7.4	0.96	0.37	3.6	0.0019	Cytochrome P450, family 4, subfamily a, polypeptide 10
*Cyp27a1*	1417590_at	8.4	0.12	0.50	3.1	0.0001	Cytochrome P450, family 27, subfamily a, polypeptide 1
*Cyp2j6*	1417952_at	8.3	0.22	0.11	2.3	0.0000	Cytochrome P450, family 2, subfamily j, polypeptide 6
*Cyp4b1*	1416194_at	9.7	0.20	0.22	2.2	0.0000	Cytochrome P450, family 2, subfamily c, polypeptide 29
*Cyp3a11*	1416809_at	11.0	0.07	0.10	1.3	0.0057	Cytochrome P450, family 3, subfamily a, polypeptide 11
							
**GSTs**							
*Mgst1*	1415897_a_at	11.1	0.05	0.08	1.3	0.0014	Microsomal glutathione S-transferase 1
*Gstp1*	1449575_a_at	11.9	0.14	0.13	-1.4	0.0027	Glutathione S-transferase, pi 1
*Gstm5*	1416842_at	7.1	0.12	0.05	-1.5	0.0002	Glutathione S-transferase, mu 5
*Gstt1*	1418186_at	7.1	0.21	0.06	-1.7	0.0001	Glutathione S-transferase, theta 1
*Gstm3*	1427473_at	5.4	0.44	0.45	-2.6	0.0006	Glutathione S-transferase, mu 3
*Gstm4*	1424835_at	4.5	0.60	0.38	-2.7	0.0009	Glutathione S-transferase, mu 4
*Gstm3*	1427474_s_at	9.2	0.36	0.79	-2.8	0.0027	Glutathione S-transferase, mu 3
							
**SULTs**							
*Sult1d1*	1418138_at	9.9	0.15	0.33	1.7	0.0017	Sulfotransferase family 1D, member 1
*Chst4*	1453393_a_at	4.7	0.04	0.08	1.4	0.0008	Carbohydrate (chondroitin 6/keratan) sulfotransferase 4
							
**AKRs**							
*Akr1b7*	1423556_at	10.3	0.04	0.63	2.5	0.0008	Aldo-keto reductase family 1, member B7

A = the average log2 transformed expression value of normal fed and 24 hours fasted mice (n = 3), SD = standard deviation (n = 3), WT = wild-type mice.

#### ABC transporters

Effects of fasting on expression of ABC transporters are summarized in Table 5. We analyzed 40 of the 52 murine ABC transporters (see additional file 3). The transporters *Abca1 *[38], involved in basolateral efflux of cholesterol, and *Abcg8 *[39], involved in transport of sterols and stanols across the apical membrane, were upregulated.

**Table 5:** Differential expressed ABC transporters in the small intestine after 24 h fasting.

Gene symbol	Affy probe set ID	A value	SD WT 0 hr	SD WT 24 hr	Fold change	P-value	Localization	Gene name
*Abca1*	1421840_at	6.266	0.28	0.44	2.3	0.0005	Basolateral (secretion)	ATP-binding cassette, sub-family A (ABC1), member 1
*Abcg8*	1420656_at	8.037	0.18	0.25	1.8	0.0003	Apical (secretion)	ATP-binding cassette, sub-family G (WHITE), member 8

A = the average log2 transformed expression value of normal fed and 24 hours fasted mice (n = 3), SD = standard deviation, WT = wild-type mice. In addition to the fold change, the intracellular localization of the corresponding protein is given.

### Time dependent changes of gene expression during fasting

**Figure 1:**
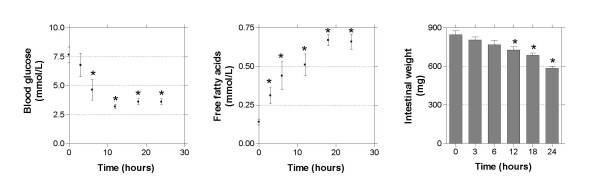
**Blood glucose levels, free fatty acid levels, and small intestinal weight during fasting**. Significance was determined using an unpaired student's *t*-test. * P-value < 0.05. Bars represent standard error.

**Figure 2:**
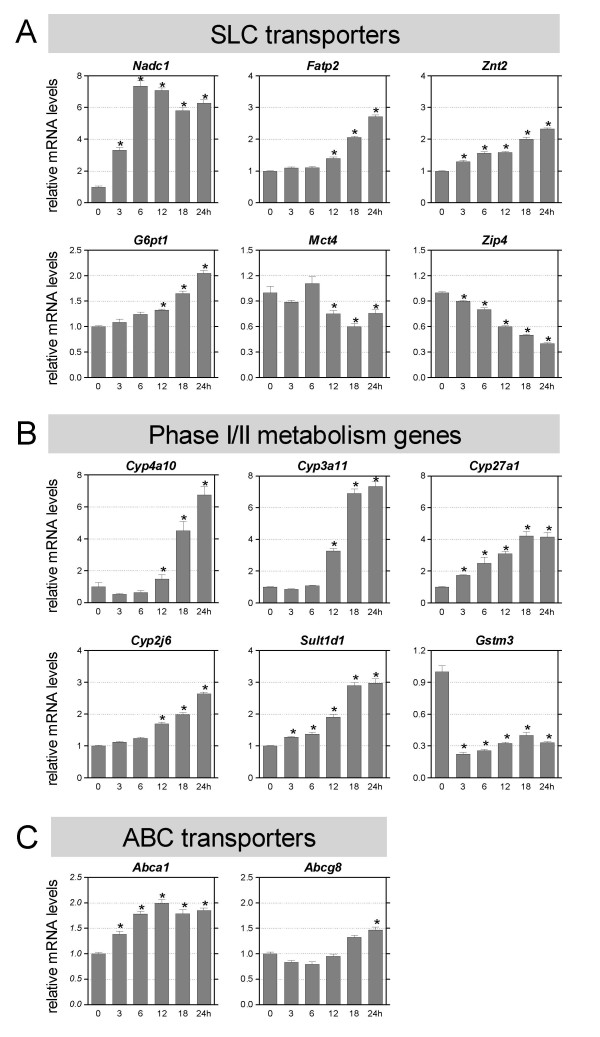
**Time dependent changes in gene expression during fasting**. The horizontal axis indicates the hours of fasting. Significance was determined using an unpaired student's *t*-test. * P-value < 0.05. Data are presented as mean ± standard error, n = 6–10. qRT-PCR results of SLC transporters. (B) qRT-PCR results of detoxification genes. (C) qRT-PCR results of ABC transporters.

To get insight into the time-dependent effects of fasting, we performed an additional experiment in which mice were deprived of food for various time points up to 24 hours. Intestinal weight, blood glucose and plasma free fatty acid levels were measured (Figure 1). As expected, blood glucose levels decreased until 12 hours of fasting, after which it stabilized [2]. Free fatty acid levels rapidly increased at the onset of fasting, and remained constant after 18 hours. Intestinal weight decreased already after 3 hours of fasting, significant changes were found as from 12 hours of fasting. For selected transporters and phase I/II metabolism enzymes gene expression was measured using qRT-PCR (Figure 2). Genes were selected based on function and array data. Several genes were regulated gradually in time and seemed to follow the same pattern as the free fatty acid concentration (*Fatp2*, *Znt2*, *G6pt1*, *Zip4*, *Cyp27a1*, *Cyp2j6*, and *Sult1d1*). Other genes were acutely regulated (*Nadc1*, *Znt2*, *Zip4*, *Cyp27a1*, *Sult1d1*, *Gstm3*, and *Abca1*.) In this group *Nadc1 *and *Gstm3 *were most drastic regulated after 3 hours fasting. Finally, a group of genes responded only after prolonged fasting (*G6pt1*, *Fatp2*, *Mct4*, *Cyp4a10*, *Cyp3a11*, *Cyp2j6*, and *Abcg8*). These data indicate that different mechanisms underlay the response to fasting.

### Role of PPARα during fasting

**Figure 3:**
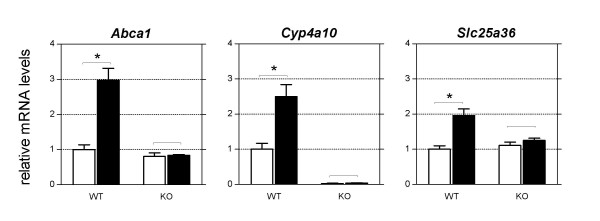
**qRT-PCR results of PPARα dependently regulated genes during fasting**. White bars represent the control group, black bars represent the 24 hours fasted group. Significance was determined using an unpaired student's *t*-test. * P-value < 0.05. Data are presented as mean ± standard error, n = 3.

It has been shown that PPARα is an important mediator of the hepatic adaptive response to fasting [2]. Therefore we evaluated the role of this transcription factor in regulating transporter and phase I/II metabolism genes in small intestine during fasting. To this end, the effect of 24 hours fasting was compared in wild-type and PPARα knockout mice (Table 6). Eight of the genes identified in wild-type mice were PPARα-dependently regulated, which corresponded to 24% of all genes regulated. No genes were found to be suppressed in a PPARα dependent manner. qRT-PCR was used to confirm the differential expression of *Cyp4a10*, *Abca1*, and *Slc25a36 *(Figure 3).

**Table 6:** PPARα regulated genes during fasting.

**Gene Symbol**	**Probe Set ID**	**FC WTc vs WT 24 hr**	**P-value WT**	**FC KOc vs KO 24 hr**	**A value WT**
*Cyp4a10*	1424853_s_at	3.6	0.0019	NC	7.4
*Abca1*	1421840_at	2.3	0.0005	NC	6.3
*Slc25a36*	1419656_at	1.7	0.0002	NC	6.7
*Slc25a36*	1419657_a_at	1.5	0.0007	NC	9.4
*Smct1, Slc5a8*	1425606_at	1.5	0.0009	NC	6.5
*Sert, Slc6a4*	1417150_at	1.4	0.0014	NC	8.5
*Dtd, Slc26a2*	1421145_at	1.4	0.0028	NC	5.6
*Chst4*	1453393_a_at	1.4	0.0008	NC	4.7
*Mgst1*	1415897_a_at	1.3	0.0014	NC	11.1

FC = Fold change, WT = wild-type mice. KO = PPARα-null mice, A = the average log2 transformed expression value of normal fed (c) and 24 hours (24 hr) fasted mice (n = 3).

## Discussion

**Figure 4:**
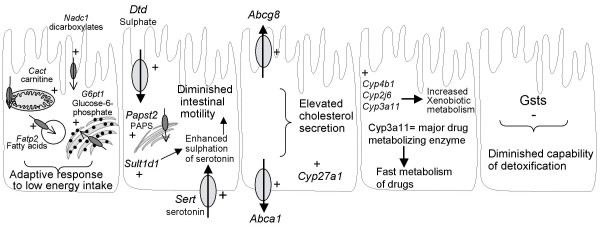
**Hypothetical schematic overview of effects of fasting on small intestinal transporters and phase I/II metabolism genes**. Not all results are summarized in this figure. The direction of transport is shown (↑) in the transporters, + means upregulated and – means downregulated during fasting. (A) Increased energy metabolism. (B) Diminished intestinal motility. (C) Elevated cholesterol secretion. (D) Increased xenobiotic metabolism. (E) Diminished capability of detoxification. (F) PPARα dependent regulated genes.

In this study we set out to determine the effect of fasting on several aspects directly related to the primary function of the small intestine; the selective absorption and metabolism of food components. We find that of all genes encoding transport proteins and phase I/II metabolic enzymes, approximately 13% were changed due to 24 hours of fasting. Effects on gene expression are dependent on the length of food deprivation, and PPARα is required for the adaptive response of a subset of genes. A summary of hypothetical functional outcomes of fasting in the murine small intestine is presented in Figure 4, and detailed below. Although in this study we only determined mRNA levels, it has been reported that for the majority of genes the mRNA levels reflect protein abundance very well [40,41]. We therefore allow ourselves to speculate about the functional consequences of fasting. Nevertheless, these implications should ultimately be evaluated in follow-up studies.

Changes in intestinal structure upon fasting differ between species [20]. Although we did not investigate this in the current study, both Lenaerts et al. [42] and Chappell et al. [43] observed only minor changes on murine mucosal structure upon fasting. In addition, if occurring, we believe that changes on tissue base do not affect our gene expression data, because otherwise we should have seen more pronounced differences on gene expression level.

An array of intestinal transporters and phase I and II metabolic enzymes are required to handle adequately various endogenous molecules, food components including nutrients and xenobiotics. Our finding that 24 hours of fasting results in the differential expression of 13% of the transporter and phase I/II metabolism genes has implications for metabolism of the different types of substrates.

### Regulation of genes involved in intestinal motility

Expression of the serotonin transporter *Sert *was increased after 24 hours of fasting. Serotonin, a neurotransmitter secreted by enterochromaffin cells, is considered to play a key role in normal functioning of the gut, initiating peristaltic reflex pathways and facilitating propulsive activity. Sert-mediated uptake of serotonin in enterocytes is responsible for the termination of the action of serotonin in the intestine [28]. After a meal, the small intestine exhibits a pattern of phasic contractions of various amplitudes [44]. During fasting, these phasic contractions are replaced by a cyclic pattern with less frequent contractions, enough to propel undigested food residues and sloughed enterocytes. As it is known that serotonin increases the frequency of intestinal contractions [45], removal of serotonin by *Sert *preserves these lower motility reflexes during fasting. *Sult1d1 *is involved in the sulfation of serotonin for the serotonin removal in enterocytes [46]. For this conjugation the activated form of sulphate, PAPS, is needed. *Sult1d1*, the apical sulphate transporter *Dtd*, and the PAPS transporter *Papst2 *were all upregulated during fasting. We believe that the coordinated induction of these enzymes is required to maintain the lower peristaltic reflexes during fasting (Figure 4B).

### Regulation of genes involved in metabolism of energy-yielding substrates

Fasting increased the expression of *Cact *and *Fatp2*, and *G6pt1*, three intracellular SLC transporters involved in transport of substrates of mitochondrial β-oxidation and glycogenolysis, respectively [30,31,33,47]. Changes in gene expression of these transporters coincided with differential gene expression of enzymes involved in both processes (data not shown). In addition, the increased expression of *Cyp4a10 *and *Cyp4b1 *points to enhanced peroxisomal oxidation of fatty acids [11], and an elevated level of *Nadc1 *implies increased uptake from luminal dicarboxylates (Krebs cycle intermediates) as well as citrate secreted by pancreatic and gastric juice [48]. Thus, these changes reflect the molecular events caused by the switch of fuel utilization from carbohydrates to fatty acid oxidation (Figure 4A).

### Regulation of genes involved in cholesterol efflux

Cholesterol can be secreted from enterocytes by chylomicrons and by the efflux transporters Abca1 and Abcg5/g8. Expression of the cholesterol efflux transporters *Abca1 *and *Abcg8 *was increased upon fasting. Since both carriers are LXR target genes [49,50] we believe this is due to the profoundly increased expression levels of Cyp27a1, which results in enhanced levels of the potent LXR agonist 27-hydroxycholesterol [51]. Since during fasting no cholesterol is required for chylomicron formation, we speculate this may be a compensatory mechanism for the enterocyte to balance its intracellular cholesterol concentrations (Figure 4C).

### Regulation of genes involved in metabolism of xenobiotics

Various CypP450s with well-known drug-metabolizing capacity were differentially expressed after fasting (*Cyp2j6*, *Cyp3a11*, and *Cyp4b1*). Increased expression of *Cyp3a11 *after fasting has also been observed in rat liver [52]. Although *Cyp3a11 *does not have a direct human ortholog, it has similar substrate specificity as human *CYP3A4*. *CYP3A4 *is considered to be the major metabolizing enzyme of approximately half of the drugs in use today [53]. Our results indicate that upon a fasting period drugs may be more efficiently detoxified (Figure 4D), which is of relevance e.g. during surgical interventions in patients.

Except for Gsts, which were expressed at reduced levels, fasting had no denoting effect on expression of phase II metabolism genes. GSH plays an important role in the defense against oxidative stress [54], and is required as cofactor for glutathione peroxidase (GPx) and glutathione S-transferase (Gst) activity. GPx detoxifies peroxides using GSH as an electron donor, producing GSSG as end product which in turn is converted back into GSH by glutathione reductase (Gsr). Gsts catalyze the conjugation of GSH to a wide variety of endogenous and exogenous electrophilic compounds. It is known that upon fasting levels of GSH are reduced in small intestine and liver [55-57], likely as a result of increased oxidation and decreased presence of its dietary precursory amino acids [58]. We believe these reduced GSH levels may be responsible for the observed reduced mRNA levels of the glutathione S-transferase (Gsts), since it is known that expression levels of Gsts are directly dependent on the presence of GSH [59]. In addition, it is known that dietary electrophiles are able to activate the transcription factor Nrf2, which can activate among others Gsts [60]. During fasting these dietary electrophiles are not available, which may be an additional explanation for the reduced expression levels of Gsts. The time dependent fasting experiment showed that the reduction of *Gstm3 *is a very acute process. Thus, regardless the underlying mechanism, the reduced GSH and Gsts levels will render the fasting gut more sensitive towards electrophilic stressors and other Gst substrates, which may have implications for humans on drug therapy during fasting and the period directly thereafter (Figure 4E).

### Role of PPARα during fasting

We showed that eight of the transporters and phase I/II metabolism genes were PPARα dependently regulated during fasting (Figure 4F). Genes that were most prominently regulated by PPARα were involved in lipid metabolism (*Cyp4a10*, *Abca1*, and *Smct1*). In liver *Cyp4a10 *and *Abca1 *are known to be regulated via PPARα during fasting [61,62]. Furthermore, *Cyp4a10 *is a known PPARα target gene [63]. It has also been reported that Abca1 is regulated by PPARα [64], although no PPAR responsive elements have been identified in its promoter region [65]. *Sert *and *Dtd *were PPARα dependently regulated, which indicates that PPARα may link nutritional status to peristaltic movement. *Sert*, *Dtd*, *Slc25a36*, *Smct1*, *Chst4*, and *Mgst1 *have all not been identified yet as PPARα target genes. We conclude that PPARα is required for the adaptive response of a subset of genes.

## Conclusion

In this study we provide an overview of the effects of fasting on expression of transporter and phase I/II metabolism genes in the small intestine. Twenty-four hours of fasting had a high impact on gene expression of murine small intestinal transporter and phase I/II metabolism genes. In addition, we demonstrated that the effects on gene expression are dependent on the length of food deprivation. Affected processes can functionally be summarized as a) increased oxidation of fat and xenobiotics, b) increased activation of PPARα, c) increased cholesterol secretion, d) increased susceptibility to electrophilic stressors, and e) reduced intestinal motility. Finally, we showed that PPARα mediates a part of the adaptive response to fasting.

## Methods

### Animals

Pure bred wild-type (129S1/SvImJ) and PPARα-null (129S4/SvJae) mice [66] were purchased from Jackson Laboratories (Bar Harbor, ME) and bred at the animal facility of Wageningen University. Mice were housed in a light- and temperature-controlled facility and had free access to water and standard laboratory chow (RMH-B, Hope Farms, Woerden, the Netherlands). All animal studies were approved by the Local Committee for Care and Use of Laboratory Animals.

### Experimental design and tissue handling

Three to four month old male PPARα-null and wild-type mice were fasted for several time points up to 24 hours. Fasting experiments started at the onset of the light cycle. Mice were anaesthetized with a mixture of isofluorane (1.5%), nitrous oxide (70%) and oxygen (30%). Blood was collected via orbital puncture, plasma was obtained by centrifuging at 200 g for 10 minutes and stored at -80°C until use. The small intestines were excised, flushed with ice-cold PBS, and weighted. Remaining fat and pancreatic tissue was carefully removed and the small intestine was snap-frozen in liquid nitrogen and stored at -80°C until RNA isolation.

### RNA isolation and quality control

Total RNA was isolated from small intestinal samples using TRIzol reagent (Invitrogen, Breda, the Netherlands) according to the manufacturer's instructions. RNA was treated with DNAse and purified using the SV total RNA isolation system (Promega, Leiden, the Netherlands). Concentrations and purity of RNA samples were determined on a NanoDrop ND-1000 spectrophotometer (Isogen, Maarssen, the Netherlands). RNA integrity was checked on an Agilent 2100 bioanalyzer (Agilent Technologies, Amsterdam, the Netherlands) with 6000 Nano Chips according to the manufacturer's instructions. RNA was judged as suitable for array hybridization only if samples exhibited intact bands corresponding to the 18S and 28S ribosomal RNA subunits, and displayed no chromosomal peaks or RNA degradation products.

### Affymetrix GeneChip oligoarray hybridization and scanning

For microarray analyses, we used RNA isolated from the full-length small intestine. RNA was hybridized on an Affymetrix GeneChip Mouse Genome 430A array. This array detects 22,690 transcripts that represent 12,453 known genes. For each experimental group, three biological replicated were hybridized, thus in total 12 arrays were used. Detailed methods for the labeling and subsequent hybridizations are available on request. Arrays were scanned on a GeneChip Scanner 3000 (Affymetrix). Array data have been submitted to the Gene Expression Omnibus, accession number GSE6864.

### Analyses of microarray data

Scans of the Affymetrix arrays were processed using packages from the Bioconductor project [67]. Expression levels of probe sets were estimated using the library GCRMA [68], where after differentially expressed probe sets were identified using linear models [69]. The library LIMMA implements an empirical Bayes method to assign differential gene expression, an approach repeatedly shown to be the most appropriate [70-73]. To compile a list of transporter and phase I/II metabolism genes present on the array, annotation information from Affymetrix (release of July 2006) was queried for SLC transporters, ABC transporters, CypP450s, the phase II metabolism enzymes glutathione S-transferases, sulfotransferases, epoxide hydrolases, aldo-keto reductases, N-acetyltransferases, and glucuronosyl transferases. Also glutathione reductase, glutathione synthetase, and glutathione peroxidases were included in this set. The final set consisted of 665 probesets, encoding for 436 unique genes (Table 1). To study significantly expressed genes, only probesets with an expression value higher than 20 in the WT control group were selected for further analysis. This filtering was done after normalization. Probe sets that satisfied the criterion of >1.3 fold change with a p-value < 0.01 were considered to be significantly regulated. Of these, probe sets that were not changed in fasted PPARα-knockout mice, were designated PPARα regulated. Interpretations of functional outcomes of fasting focused on groups of genes that are known to be functionally related (i.e. participating in the same pathway or having a similar function). Although at first sight the fold change threshold may seem to be low, we could confirm all changes in gene expression identified on the microarray (Table 2). Moreover, we would like to stress that it is generally accepted that effects of nutritional interventions on gene expression are subtle, in contrast to pharmacological-type of interventions [74-76]. A clear example of this is found in Patsouris et al. [10], in which the effect of pharmacological, physiological, and nutritional intervention on expression of the same set of genes were compared.

### Quantitative Real-time PCR

Single-stranded complementary DNA (cDNA) was synthesized from 1 μg of total RNA using the Reverse transcription system (Promega, Leiden, The Netherlands) following the supplier's protocol. cDNA was PCR amplified with Platinum Taq DNA polymerase (all reagents were from Invitrogen). Primer sequences used in the PCR reaction were chosen based on the sequences available in GenBank. The sequence of primers used is available in Table 7. PCR was carried out using SYBR green on a MyIQ thermal cycler (Bio-Rad laboratories BV, Veenendaal, The Netherlands) with the following thermal cycling conditions: 8 min at 94°C, followed by 45 cycles of 94°C for 15 s and 60°C for 1 min. All samples were performed in duplicate and normalized to cyclophilin expression.

**Table 7:** Primer sequences.

**Gene symbol**	**Forward primer**	**Reverse primer**
*Abca1*	CCCAGAGCAAAAAGCGACTC	CCCAGAGCAAAAAGCGACTC
*Abcg8*	AGTGGTCAGTCCAACACTCTG	GAGACCTCCAGGGTATCTTGAA
*Chst4*	GTCTTTGATGCCTACATGAACCC	GTGGGCAGGGAAGAAGTCA
*Cyclophilin*	CAGACGCCACTGTCGCTTT	TGTCTTTGGAACTTTGTCTGCAA
*Cyp2j6*	TTAGCCACGATCTGGGCAG	CTGGGGGATAGTTCTTGGGG
*Cyp3a11*	TGAAACCACCAGTAGCACACTT	CAGGTATTCCATCTCCATCACA
*Cyp4a10*	ACCACAATGTGCATCAAGGAGGCC	AGGAATGAGTGGCTGTGTCGGGGAGAG
*Cyp27a1*	GCCTTGCACAAGGAAGTGACT	CGCAGGGTCTCCTTAATCACA
*Dtd*	AAGAGCAGCATGACCTCTCAC	CTGCCTCAAGTCAGTGCCT
*Fatp2*	ACAACATTCGTGCCAAGTCTCT	CTCCTCCACAGCTTCTTGTAGATC
*G6pt1*	GGCTACGGCTACTATCGCAC	AGGAGGGCATGACAAAGGAGA
*Gstm3*	CCCCAACTTTGACCGAAGC	GGTGTCCATAACTTGGTTCTCCA
*Mct4*	GAGGTGGTTCATACCCCGGAAA	ATATGAGCGTTGCCCAGTCTCT
*Mgst1*	TCGCACTGACGAGAAGGTG	TGCATGAGGGCTGTAGAGAGA
*Nadc1*	TCACAGCCTTCCTCTCCATGT	ACTATTGCCTTCCTCCACATCCT
*Slc25a28*	AGCATTGCGTGATGTACCCG	CCTGTTGCTGTGACGTTCA
*Slc25a36*	GTGAACCGAGTAGTGTCCCCT	CCTTGCAGTTTGAATAAGCAGC
*Sult1d1*	ATGTCTTCAGGAGGGAGTTAGTG	CATCAGGCCGGGCTTCAAA
*Zip4*	ATGCTCCCAAAGTCGGTCAC	CAGCGTATTTAACAGGCCGTC
*Znt2*	AACTGCCAGGCGTGCCAGGG	CCGTGGAGTGGTCCAGGCTGTG

### Free fatty acids and blood glucose levels

Free fatty acids were measured with the Free fatty acids half-micro test (Roche Diagnostics, Almere, The Netherlands) according to the manufacturer's instructions. Blood glucose levels were determined by the Accu-Chek Compact Glucose (Roche Diagnostics, Almere, The Netherlands) with 1 drop of blood obtained by orbital puncture.

## Authors' contributions

MM conceived the study and supervised its design and coordination. The design of the study was set up by HB, MB, and GH. JM hybridized the microarrays and was together with HB, MB and GH involved in experimental work. Microarray analysis was performed by GH, PG, and HB. HB drafted the manuscript and GH and MM participated in its preparation. All authors have read and approved the final manuscript.

## Supplementary Material

Additional file 1 **Expression of intestinal SLC transporters after a 24 hour fasting period**. This file contains the expression data, fold changes, and p-values for all SLC transporters in fed and 24 hours fasted mice. Click here for file

Additional file 2 **Expression of intestinal detoxification enzymes after a 24 hour fasting period**. This file contains the expression data, fold changes, and p-values for all detoxification enzymes in fed and 24 hours fasted mice. Click here for file

Additional file 3 **Expression of intestinal ABC transporters after a 24 hour fasting period**. This file contains the expression data, fold changes, and p-values for all ABC transporters in fed and 24 hours fasted mice. Click here for file
