# Supplementary material for: Gene expression of transporters and phase I/II metabolic enzymes in murine small intestine during fasting
Source: BMC Genomics. 2007 Aug 7;8:267. doi: 10.1186/1471-2164-8-267 (PMC1971072; doi:10.1186/1471-2164-8-267)
Supplement: Additional file 1 — Expression of intestinal SLC transporters after a 24 hour fasting period. This file contains the expression data, fold changes, and p-values for all SLC transporters in fed and 24 hours fasted mice. [file 1471-2164-8-267-S1.pdf]

Gene Symbol	Probe set ID	Expression signal normal fed mice	Expression signal 24h fasted mice	Fold change	P-value	Gene name
**Slc1**						
Slc1a1	1425415_a_at	6.527	6.268	0.836	0.1199	solute carrier family 1 (neuronal/epithelial high affinity glutamate transporter), member 1
Slc1a1	1448299_at	6.640	6.508	0.912	0.2785	solute carrier family 1 (neuronal/epithelial high affinity glutamate transporter), member 1
Slc1a1	1460386_a_at	4.671	4.372	0.813	0.2138	solute carrier family 1 (neuronal/epithelial high affinity glutamate transporter), member 1
Slc1a2	1451627_a_at	1.866	1.944	1.060	0.3715	solute carrier family 1 (glial high affinity glutamate transporter), member 2
Slc1a3	1426340_at	2.624	2.586	0.974	0.7046	solute carrier family 1 (glial high affinity glutamate transporter), member 3
Slc1a3	1426341_at	1.912	1.984	1.050	0.5286	solute carrier family 1 (glial high affinity glutamate transporter), member 3
Slc1a3	1452031_at	2.575	2.679	1.080	0.5221	solute carrier family 1 (glial high affinity glutamate transporter), member 3
Slc1a4	1423549_at	2.067	1.916	0.901	0.1471	solute carrier family 1 (glutamate/neutral amino acid transporter), member 4
Slc1a4	1423550_at	3.071	2.693	0.769	0.0128	solute carrier family 1 (glutamate/neutral amino acid transporter), member 4
Slc1a4	1456003_a_at	3.966	3.912	0.963	0.6459	solute carrier family 1 (glutamate/neutral amino acid transporter), member 4
Slc1a5	1416629_at	3.849	3.577	0.828	0.0285	solute carrier family 1 (neutral amino acid transporter), member 5
Slc1a6	1418933_at	2.021	2.117	1.070	0.3124	solute carrier family 1 (high affinity aspartate/glutamate transporter), member 6
**Slc2**						
Slc2a1	1426599_a_at	4.898	4.806	0.938	0.5232	solute carrier family 2 (facilitated glucose transporter), member 1
Slc2a1	1426600_at	3.471	3.239	0.852	0.0605	solute carrier family 2 (facilitated glucose transporter), member 1
Slc2a1	1434773_a_at	5.318	5.123	0.873	0.2519	solute carrier family 2 (facilitated glucose transporter), member 1
Slc2a2	1449067_at	9.868	9.948	1.060	0.6155	solute carrier family 2 (facilitated glucose transporter), member 2
Slc2a3	1421924_at	1.913	2.080	1.120	0.2696	solute carrier family 2 (facilitated glucose transporter), member 3
Slc2a3	1427770_a_at	1.818	1.824	1.000	0.9586	solute carrier family 2 (facilitated glucose transporter), member 3
Slc2a3	1437052_s_at	2.710	2.759	1.030	0.7785	solute carrier family 2 (facilitated glucose transporter), member 3
Slc2a3	1455898_x_at	2.524	2.569	1.030	0.6609	solute carrier family 2 (facilitated glucose transporter), member 3
Slc2a4	1415958_at	3.182	2.900	0.822	0.0119	solute carrier family 2 (facilitated glucose transporter), member 4
Slc2a4	1415959_at	2.063	1.768	0.815	0.0103	solute carrier family 2 (facilitated glucose transporter), member 4
Slc2a5	1416639_at	8.368	8.700	1.260	0.2334	solute carrier family 2 (facilitated glucose transporter), member 5
Slc2a8	1450628_at	3.136	3.195	1.040	0.6003	solute carrier family 2, (facilitated glucose transporter), member 8
Slc2a9	1426568_at	4.067	4.151	1.060	0.4369	solute carrier family 2 (facilitated glucose transporter), member 9
Slc2a10	1419434_at	2.547	2.631	1.060	0.5784	solute carrier family 2 (facilitated glucose transporter), member 10
Slc2a10	1449477_s_at	2.554	2.407	0.903	0.2329	solute carrier family 2 (facilitated glucose transporter), member 10
**Slc3**						
Slc3a1	1448741_at	9.371	9.408	1.030	0.7365	solute carrier family 3, member 1
Slc3a2	1425364_a_at	9.933	10.082	1.110	0.1406	solute carrier family 3 (activators of dibasic and neutral amino acid transport), member 2

Gene Symbol	Probe set ID	Expression signal 0h	Expression signal 24h	Fold change	P-value	Gene name
**Slc4**						
Slc4a1	1416464_at	3.332	3.318	0.990	0.9142	solute carrier family 4 (anion exchanger), member 1
Slc4a1	1431743_a_at	1.917	1.937	1.010	0.8208	solute carrier family 4 (anion exchanger), member 1
Slc4a1	1434502_x_at	3.180	3.113	0.955	0.6510	solute carrier family 4 (anion exchanger), member 1
Slc4a1ap	1437758_a_at	3.471	3.433	0.974	0.7590	solute carrier family 4 (anion exchanger), member 1, adaptor protein
Slc4a2	1416637_at	6.012	5.815	0.872	0.2008	solute carrier family 4 (anion exchanger), member 2
Slc4a3	1418485_at	2.106	1.893	0.863	0.0403	solute carrier family 4 (anion exchanger), member 3
Slc4a4	1421225_a_at	5.657	5.894	1.180	0.1337	solute carrier family 4 (anion exchanger), member 4
Slc4a4	1426432_a_at	4.503	4.752	1.190	0.0781	solute carrier family 4 (anion exchanger), member 4
Slc4a4	1450169_at	1.788	1.823	1.020	0.7131	solute carrier family 4 (anion exchanger), member 4
Slc4a4	1452071_at	7.518	7.743	1.170	0.0629	solute carrier family 4 (anion exchanger), member 4
Slc4a7	1438673_at	9.538	9.563	1.020	0.8956	solute carrier family 4, sodium bicarbonate cotransporter, member 7
Slc4a8	1419851_at	2.898	2.885	0.991	0.9055	solute carrier family 4 (anion exchanger), member 8
Slc4a8	1423443_at	2.004	2.025	1.020	0.8567	solute carrier family 4 (anion exchanger), member 8
Slc4a10	1417672_at	1.963	1.718	0.844	0.0890	solute carrier family 4, sodium bicarbonate cotransporter-like, member 10
**Slc5**						
Slc5a1	1419057_at	11.626	11.857	1.170	0.0816	solute carrier family 5 (sodium/glucose cotransporter), member 1
Slc5a1	1455431_at	10.489	10.856	1.290	0.0697	solute carrier family 5 (sodium/glucose cotransporter), member 1
Slc5a2	1419166_at	5.819	5.644	0.886	0.3336	solute carrier family 5 (sodium/glucose cotransporter), member 2
Slc5a2	1455005_s_at	3.170	3.098	0.951	0.4392	solute carrier family 5 (sodium/glucose cotransporter), member 2
Slc5a3	1422170_at	2.227	2.120	0.929	0.3283	solute carrier family 5 (inositol transporters), member 3
Slc5a4a	1421637_at	6.416	5.979	0.739	0.1692	solute carrier family 5, member 4a
Slc5a4b	1422757_at	9.733	9.459	0.827	0.2622	solute carrier family 5 (neutral amino acid transporters, system A), member 4b
Slc5a5	1422113_at	2.423	2.460	1.030	0.7198	solute carrier family 5 (sodium iodide symporter), member 5
Slc5a6	1435860_at	5.030	4.682	0.786	0.0266	solute carrier family 5 (sodium-dependent vitamin transporter), member 6
Slc5a7	1421428_at	1.811	1.683	0.915	0.2347	solute carrier family 5 (choline transporter), member 7
Slc5a8	1425606_at	6.221	6.756	1.450	0.0009	solute carrier family 5 (iodide transporter), member 8
Slc5a9	1426634_at	4.118	4.037	0.945	0.5437	solute carrier family 5 (sodium/glucose cotransporter), member 9
Slc5a9	1452136_at	4.000	3.754	0.843	0.0458	solute carrier family 5 (sodium/glucose cotransporter), member 9
Slc5a11	1428752_at	6.003	5.897	0.929	0.5533	solute carrier family 5 (sodium/glucose cotransporter), member 11
**Slc6**						
Slc6a1	1452142_at	2.428	2.399	0.980	0.7795	solute carrier family 6 (neurotransmitter transporter, GABA), member 1
Slc6a2	1421641_at	2.332	2.289	0.971	0.6980	solute carrier family 6 (neurotransmitter transporter, noradrenalin), member 2
Slc6a3	1417415_at	3.065	3.271	1.150	0.1910	solute carrier family 6 (neurotransmitter transporter, dopamine), member 3

Gene Symbol	Probe set ID	Expression signal 0h	Expression signal 24h	Fold change	P-value	Gene name
Slc6a4	1417150_at	8.237	8.755	1.430	0.0014	solute carrier family 6 (neurotransmitter transporter, serotonin), member 4
Slc6a5	1425992_at	2.387	2.423	1.020	0.7212	solute carrier family 6 (neurotransmitter transporter, glycine), member 5
Slc6a6	1420148_at	3.380	3.423	1.030	0.6185	solute carrier family 6 (neurotransmitter transporter, taurine), member 6
Slc6a6	1421346_a_at	4.860	5.203	1.270	0.0192	solute carrier family 6 (neurotransmitter transporter, taurine), member 6
Slc6a6	1449751_at	2.955	2.882	0.951	0.5225	solute carrier family 6 (neurotransmitter transporter, taurine), member 6
Slc6a8	1417116_at	10.264	10.549	1.220	0.0165	solute carrier family 6 (neurotransmitter transporter, creatine), member 8
Slc6a8	1448596_at	10.030	10.184	1.110	0.1694	solute carrier family 6 (neurotransmitter transporter, creatine), member 8
Slc6a9	1417636_at	3.568	3.551	0.988	0.8975	solute carrier family 6 (neurotransmitter transporter, glycine), member 9
Slc6a9	1431812_a_at	2.772	2.742	0.980	0.7497	solute carrier family 6 (neurotransmitter transporter, glycine), member 9
Slc6a12	1449382_at	2.078	2.041	0.974	0.8119	solute carrier family 6 (neurotransmitter transporter, betaine/GABA), member 12
Slc6a13	1424338_at	1.769	1.894	1.090	0.2906	solute carrier family 6 (neurotransmitter transporter, GABA), member 13
Slc6a14	1420503_at	3.000	2.989	0.992	0.9170	solute carrier family 6 (neurotransmitter transporter), member 14
Slc6a14	1420504_at	2.787	2.814	1.020	0.8944	solute carrier family 6 (neurotransmitter transporter), member 14
Slc6a15	1426712_at	2.559	2.690	1.100	0.1974	solute carrier family 6 (neurotransmitter transporter), member 15
Slc6a17	1436137_at	2.450	2.504	1.040	0.6036	solute carrier family 6 (neurotransmitter transporter), member 17
Slc6a18	1451879_a_at	2.846	3.114	1.200	0.0339	solute carrier family 6 (neurotransmitter transporter), member 18
Slc6a20	1422899_at	2.104	2.047	0.961	0.5742	solute carrier family 6 (neurotransmitter transporter), member 20
**Slc7**						
Slc7a1	1421533_at	2.178	2.087	0.939	0.4081	solute carrier family 7 (cationic amino acid transporter, y+ system), member 1
Slc7a2	1422648_at	2.220	2.178	0.971	0.6983	solute carrier family 7 (cationic amino acid transporter, y+ system), member 2
Slc7a2	1426008_a_at	2.101	1.958	0.906	0.1795	solute carrier family 7 (cationic amino acid transporter, y+ system), member 2
Slc7a2	1450703_at	2.669	2.569	0.933	0.3374	solute carrier family 7 (cationic amino acid transporter, y+ system), member 2
Slc7a3	1417022_at	1.697	1.759	1.040	0.5518	solute carrier family 7 (cationic amino acid transporter, y+ system), member 3
Slc7a4	1426068_at	2.240	2.079	0.894	0.1550	solute carrier family 7 (cationic amino acid transporter, y+ system), member 4
Slc7a4	1426069_s_at	2.583	2.555	0.981	0.7852	solute carrier family 7 (cationic amino acid transporter, y+ system), member 4
Slc7a4	1436776_x_at	2.681	2.724	1.030	0.7821	solute carrier family 7 (cationic amino acid transporter, y+ system), member 4
Slc7a5	1418326_at	3.266	3.283	1.010	0.8482	solute carrier family 7 (cationic amino acid transporter, y+ system), member 5
Slc7a6	1433467_at	2.713	2.586	0.916	0.3237	solute carrier family 7 (cationic amino acid transporter, y+ system), member 6
Slc7a6	1460541_at	2.624	2.618	0.996	0.9478	solute carrier family 7 (cationic amino acid transporter, y+ system), member 6
Slc7a7	1417392_a_at	9.628	9.993	1.290	0.0083	solute carrier family 7 (cationic amino acid transporter, y+ system), member 7
Slc7a8	1417929_at	5.701	5.817	1.080	0.4508	solute carrier family 7 (cationic amino acid transporter, y+ system), member 8
Slc7a9	1448783_at	10.394	10.534	1.100	0.1627	solute carrier family 7 (cationic amino acid transporter, y+ system), member 9
Slc7a10	1421093_at	1.856	1.801	0.963	0.5671	solute carrier family 7 (cationic amino acid transporter, y+ system), member 10
Slc7a11	1420413_at	2.088	1.953	0.911	0.2156	solute carrier family 7 (cationic amino acid transporter, y+ system), member 11

Gene Symbol	Probe set ID	Expression signal 0h	Expression signal 24h	Fold change	P-value	Gene name
Slc7a12	1419579_at	1.680	1.703	1.020	0.8176	solute carrier family 7 (cationic amino acid transporter, y+ system), member 12
Slc7a13	1431740_at	2.034	1.967	0.954	0.4834	solute carrier family 7, (cationic amino acid transporter, y+ system) member 13
Slc7a13	1449301_at	2.348	2.338	0.993	0.9260	solute carrier family 7, (cationic amino acid transporter, y+ system) member 13
**Slc8**						
Slc8a1	1425817_a_at	2.480	2.443	0.975	0.7383	solute carrier family 8 (sodium/calcium exchanger), member 1
Slc8a2	1428054_at	2.014	2.029	1.010	0.8773	solute carrier family 8 (sodium/calcium exchanger), member 2
Slc8a3	1450311_at	1.676	1.661	0.989	0.8720	solute carrier family 8 (sodium/calcium exchanger), member 3
**Slc9**						
Slc9a1	1417397_at	6.476	6.187	0.818	0.0291	solute carrier family 9 (sodium/hydrogen exchanger), member 1
Slc9a3r1	1438115_a_at	10.613	10.356	0.837	0.0876	solute carrier family 9 (sodium/hydrogen exchanger), isoform 3 regulator 1
Slc9a3r1	1438116_x_at	10.624	10.517	0.928	0.2801	solute carrier family 9 (sodium/hydrogen exchanger), isoform 3 regulator 1
Slc9a3r1	1450982_at	10.868	10.799	0.954	0.5759	solute carrier family 9 (sodium/hydrogen exchanger), isoform 3 regulator 1
Slc9a3r2	1428954_at	3.329	3.125	0.868	0.0867	solute carrier family 9 (sodium/hydrogen exchanger), isoform 3 regulator 2
Slc9a3r2	1428955_x_at	3.172	3.159	0.991	0.8868	solute carrier family 9 (sodium/hydrogen exchanger), isoform 3 regulator 2
Slc9a3r2	1431208_a_at	3.063	2.986	0.948	0.4057	solute carrier family 9 (sodium/hydrogen exchanger), isoform 3 regulator 2
Slc9a3r2	1439368_a_at	3.654	3.351	0.811	0.0229	solute carrier family 9 (sodium/hydrogen exchanger), isoform 3 regulator 2
Slc9a3r2	1439369_x_at	2.612	2.436	0.885	0.0650	solute carrier family 9 (sodium/hydrogen exchanger), isoform 3 regulator 2
Slc9a3r2	1452976_a_at	2.182	2.111	0.952	0.4551	solute carrier family 9 (sodium/hydrogen exchanger), isoform 3 regulator 2
Slc9a8	1426274_at	3.980	4.059	1.060	0.4652	solute carrier family 9 (sodium/hydrogen exchanger), member 8
Slc9a8	1454865_at	3.073	3.166	1.070	0.5040	solute carrier family 9 (sodium/hydrogen exchanger), member 8
**Slc10**						
Slc10a1	1424898_at	2.345	2.369	1.020	0.8358	solute carrier family 10 (sodium/bile acid cotransporter family), member 1
Slc10a1	1450261_a_at	1.552	1.570	1.010	0.8493	solute carrier family 10 (sodium/bile acid cotransporter family), member 1
Slc10a2	1450245_at	4.692	4.765	1.050	0.5680	solute carrier family 10, member 2
Slc10a3	1451227_a_at	4.263	4.294	1.020	0.7832	solute carrier family 10 (sodium/bile acid cotransporter family), member 3
Slc10a6	1428776_at	2.562	2.531	0.979	0.7912	solute carrier family 10 (sodium/bile acid cotransporter family), member 6
**Slc11**						
Slc11a1	1420361_at	3.680	3.391	0.818	0.1043	solute carrier family 11 (proton-coupled divalent metal ion transporters), member 1
Slc11a2	1417584_at	3.618	3.665	1.030	0.6520	solute carrier family 11 (proton-coupled divalent metal ion transporters), member 2
Slc11a2	1426441_at	4.862	4.468	0.761	0.0962	solute carrier family 11 (proton-coupled divalent metal ion transporters), member 2
Slc11a2	1452078_a_at	4.912	4.597	0.804	0.0825	solute carrier family 11 (proton-coupled divalent metal ion transporters), member 2
**Slc12**						
Slc12a1	1421390_at	2.864	2.890	1.020	0.8538	solute carrier family 12, member 1
Slc12a1	1424260_at	2.211	2.159	0.964	0.6157	solute carrier family 12, member 1

Gene Symbol	Probe set ID	Expression signal 0h	Expression signal 24h	Fold change	P-value	Gene name
Slc12a2	1417622_at	9.192	9.224	1.020	0.7406	solute carrier family 12, member 2
Slc12a2	1417623_at	5.794	6.072	1.210	0.1522	solute carrier family 12, member 2
Slc12a2	1448780_at	4.787	5.014	1.170	0.0699	solute carrier family 12, member 2
Slc12a3	1422856_at	2.690	2.718	1.020	0.7972	solute carrier family 12, member 3
Slc12a4	1417446_at	3.436	3.372	0.956	0.5672	solute carrier family 12, member 4
Slc12a5	1425337_at	2.246	2.221	0.983	0.8376	solute carrier family 12, member 5
Slc12a5	1451674_at	2.232	2.102	0.914	0.2609	solute carrier family 12, member 5
Slc12a6	1436989_s_at	1.366	1.772	1.330	0.0453	solute carrier family 12, member 6
Slc12a6	1449878_a_at	5.654	5.950	1.230	0.0229	solute carrier family 12, member 6
Slc12a7	1418257_at	8.032	8.191	1.120	0.1105	solute carrier family 12, member 7
Slc12a7	1449011_at	4.900	4.658	0.845	0.3811	solute carrier family 12, member 7
Slc12a8	1420334_at	5.870	5.994	1.090	0.4407	solute carrier family 12 (potassium/chloride transporters), member 8
Slc12a9	1418103_at	3.952	4.156	1.150	0.1798	solute carrier family 12 (potassium/chloride transporters), member 9
**Slc13**						
Slc13a1	1431379_a_at	8.949	8.963	1.010	0.9515	solute carrier family 13 (sodium/sulphate symporters), member 1
Slc13a2	1418857_at	9.891	10.548	1.580	0.0011	solute carrier family 13 (sodium-dependent dicarboxylate transporter), member 2
Slc13a3	1416560_at	2.671	2.666	0.996	0.9574	solute carrier family 13 (sodium-dependent dicarboxylate transporter), member 3
Slc13a3	1438377_x_at	3.822	3.860	1.030	0.7634	solute carrier family 13 (sodium-dependent dicarboxylate transporter), member 3
Slc13a3	1439384_at	1.496	1.388	0.928	0.2547	solute carrier family 13 (sodium-dependent dicarboxylate transporter), member 3
Slc13a3	1439385_x_at	1.575	1.544	0.979	0.8153	solute carrier family 13 (sodium-dependent dicarboxylate transporter), member 3
**Slc14**						
Slc14a1	1428114_at	3.163	3.114	0.966	0.6623	solute carrier family 14 (urea transporter), member 1
Slc14a1	1448640_at	2.760	2.789	1.020	0.8644	solute carrier family 14 (urea transporter), member 1
Slc14a2	1425250_a_at	1.918	1.836	0.945	0.3866	solute carrier family 14 (urea transporter), member 2
Slc14a2	1426109_a_at	2.403	2.348	0.963	0.6275	solute carrier family 14 (urea transporter), member 2
**Slc15**						
Slc15a1	1419343_at	8.277	8.163	0.924	0.4183	solute carrier family 15 (oligopeptide transporter), member 1
Slc15a2	1417600_at	2.780	3.262	1.400	0.2628	solute carrier family 15 (H+/peptide transporter), member 2
Slc15a2	1424730_a_at	2.239	2.184	0.962	0.6085	solute carrier family 15 (H+/peptide transporter), member 2
Slc15a3	1420697_at	3.038	2.776	0.834	0.0291	solute carrier family 15, member 3
Slc15a4	1448385_at	5.565	5.396	0.890	0.1494	solute carrier family 15, member 4
**Slc16**						
Slc16a1	1415802_at	9.027	9.155	1.090	0.2613	solute carrier family 16 (monocarboxylic acid transporters), member 1
Slc16a2	1418445_at	2.771	2.647	0.918	0.2485	solute carrier family 16 (monocarboxylic acid transporters), member 2

Gene Symbol	Probe set ID	Expression signal 0h	Expression signal 24h	Fold change	P-value	Gene name
Slc16a2	1418446_at	2.128	2.070	0.961	0.6803	solute carrier family 16 (monocarboxylic acid transporters), member 2
Slc16a3	1449005_at	4.562	3.801	0.590	0.0058	solute carrier family 16 (monocarboxylic acid transporters), member 3
Slc16a4	1426082_a_at	1.267	1.285	1.010	0.8571	solute carrier family 16 (monocarboxylic acid transporters), member 4
Slc16a6	1417884_at	3.947	4.101	1.110	0.4650	solute carrier family 16 (monocarboxylic acid transporters), member 6
Slc16a7	1448502_at	2.597	2.683	1.060	0.5845	solute carrier family 16 (monocarboxylic acid transporters), member 7
Slc16a8	1420445_at	1.710	1.803	1.070	0.3591	solute carrier family 16 (monocarboxylic acid transporters), member 8
Slc16a11	1427107_at	2.489	2.531	1.030	0.6491	solute carrier family 16 (monocarboxylic acid transporters), member 11
**Slc17**						
Slc17a1	1417280_at	2.388	2.486	1.070	0.3902	solute carrier family 17 (sodium phosphate), member 1
Slc17a2	1425033_at	2.022	2.017	0.997	0.9601	solute carrier family 17 (sodium phosphate), member 2
Slc17a2	1425034_at	2.894	2.958	1.050	0.6130	solute carrier family 17 (sodium phosphate), member 2
Slc17a3	1418923_at	1.726	1.692	0.976	0.7650	solute carrier family 17 (sodium phosphate), member 3
Slc17a6	1418610_at	1.844	1.872	1.020	0.8214	solute carrier family 17 (sodium-dependent inorganic phosphate cotransporter), member 6
Slc17a6	1428379_at	2.711	2.668	0.971	0.7183	solute carrier family 17 (sodium-dependent inorganic phosphate cotransporter), member 6
**Slc18**						
Slc18a1	1426595_at	3.004	3.089	1.060	0.3988	solute carrier family 18 (vesicular monoamine), member 1
Slc18a3	1422203_at	0.836	0.859	1.020	0.7866	solute carrier family 18 (vesicular monoamine), member 3
**Slc19**						
Slc19a1	1420138_at	4.352	4.449	1.070	0.6121	solute carrier family 19 (sodium/hydrogen exchanger), member 1
Slc19a1	1448132_at	3.602	3.663	1.040	0.7546	solute carrier family 19 (sodium/hydrogen exchanger), member 1
Slc19a1	1449744_at	2.010	1.958	0.965	0.6073	solute carrier family 19 (sodium/hydrogen exchanger), member 1
Slc19a2	1417902_at	4.748	5.024	1.210	0.0762	solute carrier family 19 (thiamine transporter), member 2
Slc19a2	1426117_a_at	2.625	2.873	1.190	0.1327	solute carrier family 19 (thiamine transporter), member 2
Slc19a3	1450348_at	2.510	2.346	0.893	0.1159	solute carrier family 19 (sodium/hydrogen exchanger), member 3
**Slc20**						
Slc20a1	1438824_at	3.469	3.339	0.914	0.5576	solute carrier family 20, member 1
Slc20a1	1448568_a_at	7.383	6.996	0.764	0.2663	solute carrier family 20, member 1
Slc20a2	1434235_at	3.372	3.580	1.150	0.0551	solute carrier family 20, member 2
Slc20a2	1451768_a_at	3.200	3.204	1.000	0.9668	solute carrier family 20, member 2
**Slc21**						
Slco1a1	1420379_at	2.386	2.318	0.954	0.4816	solute carrier organic anion transporter family, member 1a1
Slco1a1	1449844_at	3.053	3.043	0.993	0.9319	solute carrier organic anion transporter family, member 1a1
Slco1a4	1420405_at	2.919	2.748	0.888	0.1569	solute carrier organic anion transporter family, member 1a4
Slco1a5	1427403_at	3.062	3.002	0.959	0.5920	solute carrier organic anion transporter family, member 1a5

Gene Symbol	Probe set ID	Expression signal 0h	Expression signal 24h	Fold change	P-value	Gene name
Slco1a5	1449203_at	2.004	2.059	1.040	0.5941	solute carrier organic anion transporter family, member 1a5
Slco1a6	1419496_at	1.777	1.708	0.953	0.5460	solute carrier organic anion transporter family, member 1a6
Slco1b2	1427825_at	2.092	2.180	1.060	0.4521	solute carrier organic anion transporter family, member 1b2
Slco1b2	1427826_a_at	2.425	2.545	1.090	0.3881	solute carrier organic anion transporter family, member 1b2
Slco1b2	1449394_at	2.003	1.870	0.912	0.1548	solute carrier organic anion transporter family, member 1b2
Slco1b2	1452494_s_at	2.157	2.461	1.230	0.1039	solute carrier organic anion transporter family, member 1b2
Slco1b2	1452569_at	2.320	2.129	0.876	0.0709	solute carrier organic anion transporter family, member 1b2
Slco1c1	1423343_at	1.965	2.090	1.090	0.4104	solute carrier organic anion transporter family, member 1c1
Slco2a1	1420913_at	8.063	8.520	1.370	0.0023	solute carrier organic anion transporter family, member 2a1
Slco2a1	1420914_at	2.514	2.583	1.050	0.5519	solute carrier organic anion transporter family, member 2a1
Slco2a1	1450032_at	6.650	6.928	1.210	0.0600	solute carrier organic anion transporter family, member 2a1
Slco3a1	1418030_at	3.493	3.506	1.010	0.9293	solute carrier organic anion transporter family, member 3a1
Slco3a1	1434537_at	3.067	3.172	1.080	0.3653	solute carrier organic anion transporter family, member 3a1
Slco3a1	1448918_at	3.602	3.556	0.968	0.7535	solute carrier organic anion transporter family, member 3a1
Slco5a1	1440874_at	2.102	2.045	0.961	0.5039	solute carrier organic anion transporter family, member 5a1
Slco6b1	1419535_at	2.037	1.864	0.887	0.0862	solute carrier organic anion transporter family, member 6b1
Slco6c1	1459092_at	3.138	3.324	1.140	0.1426	solute carrier organic anion transporter family, member 6c1
**Slc22**						
Slc22a1	1418118_at	8.471	8.835	1.290	0.0093	solute carrier family 22 (organic cation transporter), member 1
Slc22a2	1419117_at	1.551	1.551	1.000	0.9976	solute carrier family 22 (organic cation transporter), member 2
Slc22a3	1420444_at	2.418	2.868	1.370	0.0019	solute carrier family 22 (organic cation transporter), member 3
Slc22a4	1417639_at	4.538	4.600	1.040	0.6462	solute carrier family 22 (organic cation transporter), member 4
Slc22a5	1421848_at	6.375	6.589	1.160	0.4539	solute carrier family 22 (organic cation transporter), member 5
Slc22a5	1450395_at	8.523	8.558	1.020	0.8513	solute carrier family 22 (organic cation transporter), member 5
Slc22a6	1417072_at	2.465	2.298	0.891	0.1177	solute carrier family 22 (organic anion transporter), member 6
Slc22a6	1438332_at	2.794	2.793	0.999	0.9940	solute carrier family 22 (organic anion transporter), member 6
Slc22a7	1451460_a_at	2.562	2.555	0.995	0.9431	solute carrier family 22 (organic anion transporter), member 7
Slc22a8	1416966_at	2.361	2.364	1.000	0.9678	solute carrier family 22 (organic anion transporter), member 8
Slc22a8	1435418_at	2.081	1.999	0.945	0.4236	solute carrier family 22 (organic anion transporter), member 8
Slc22a12	1422897_at	2.633	2.744	1.080	0.4527	solute carrier family 22 (organic anion/cation transporter), member 12
Slc22a12	1422898_s_at	2.374	2.403	1.020	0.7495	solute carrier family 22 (organic anion/cation transporter), member 12
Slc22a13	1419129_at	1.575	1.514	0.958	0.5933	solute carrier family 22 (organic cation transporter), member 13
Slc22a17	1448209_a_at	2.724	2.758	1.020	0.7732	solute carrier family 22 (organic cation transporter), member 17
Slc22a18	1417809_at	7.383	7.094	0.818	0.0817	solute carrier family 22 (organic cation transporter), member 18

Gene Symbol	Probe set ID	Expression signal 0h	Expression signal 24h	Fold change	P-value	Gene name
Slc22a18	1427531_a_at	4.717	4.582	0.910	0.3740	solute carrier family 22 (organic cation transporter), member 18
Slc22a19	1425038_at	2.367	2.253	0.924	0.3890	solute carrier family 22 (organic anion transporter), member 19
Slc22a21	1449215_at	3.706	3.860	1.110	0.4379	solute carrier family 22 (organic cation transporter), member 21
**Slc23**						
Slc23a1	1421912_at	6.457	6.810	1.280	0.0822	solute carrier family 23 (nucleobase transporters), member 1
Slc23a1	1450404_at	3.184	3.178	0.996	0.9499	solute carrier family 23 (nucleobase transporters), member 1
Slc23a2	1417329_at	6.400	6.583	1.130	0.0745	solute carrier family 23 (nucleobase transporters), member 2
Slc23a2	1417330_at	2.974	3.101	1.090	0.4692	solute carrier family 23 (nucleobase transporters), member 2
Slc23a3	1460042_at	1.942	2.074	1.100	0.1670	solute carrier family 23 (nucleobase transporters), member 3
**Slc24**						
Slc24a1	1451647_at	1.973	1.923	0.966	0.5997	solute carrier family 24 (sodium/potassium/calcium exchanger), member 1
Slc24a3	1424308_at	5.084	4.725	0.779	0.2444	solute carrier family 24 (sodium/potassium/calcium exchanger), member 3
Slc24a6	1417811_at	3.043	3.042	0.999	0.9913	solute carrier family 24 (sodium/potassium/calcium exchanger), member 6
**Slc25**						
Slc25a2	1425013_at	1.846	1.746	0.933	0.3231	solute carrier family 25 (mitochondrial carrier, ornithine transporter) member 2
Slc25a3	1416300_a_at	11.725	11.686	0.973	0.6842	solute carrier family 25 (mitochondrial carrier, phosphate carrier), member 3
Slc25a4	1424562_a_at	9.173	9.102	0.951	0.6075	solute carrier family 25 (mitochondrial carrier, adenine nucleotide translocator), member 4
Slc25a4	1424563_at	1.748	1.741	0.995	0.9506	solute carrier family 25 (mitochondrial carrier, adenine nucleotide translocator), member 4
Slc25a4	1434897_a_at	7.625	7.756	1.100	0.4203	solute carrier family 25 (mitochondrial carrier, adenine nucleotide translocator), member 4
Slc25a4	1455069_x_at	8.560	8.532	0.981	0.8589	solute carrier family 25 (mitochondrial carrier, adenine nucleotide translocator), member 4
Slc25a5	1423772_x_at	12.276	12.327	1.040	0.5806	solute carrier family 25 (mitochondrial carrier, adenine nucleotide translocator), member 5
Slc25a5	1434801_x_at	11.458	11.504	1.030	0.6144	solute carrier family 25 (mitochondrial carrier, adenine nucleotide translocator), member 5
Slc25a5	1436874_x_at	12.416	12.581	1.120	0.1053	solute carrier family 25 (mitochondrial carrier, adenine nucleotide translocator), member 5
Slc25a5	1438360_x_at	10.020	10.255	1.180	0.1472	solute carrier family 25 (mitochondrial carrier, adenine nucleotide translocator), member 5
Slc25a5 ///	1438545_at	8.802	9.016	1.160	0.0429	solute carrier family 25 (mitochondrial carrier, adenine nucleotide translocator), member 5
Slc25a5 ///	1438546_x_at	9.826	10.010	1.140	0.0566	solute carrier family 25 (mitochondrial carrier, adenine nucleotide translocator), member 5
Slc25a5 ///	1438922_x_at	10.062	10.346	1.220	0.1703	solute carrier family 25 (mitochondrial carrier, adenine nucleotide translocator), member 5
Slc25a5 ///	1430542_a_at	12.209	12.334	1.090	0.2471	solute carrier family 25 (mitochondrial carrier, adenine nucleotide translocator), member 5
Slc25a10	1416954_at	9.082	8.847	0.850	0.1175	solute carrier family 25 (mitochondrial carrier, dicarboxylate transporter), member 10
Slc25a10	1416955_at	7.251	6.922	0.796	0.0850	solute carrier family 25 (mitochondrial carrier, dicarboxylate transporter), member 10
Slc25a11	1426586_at	7.792	7.821	1.020	0.7918	solute carrier family 25 (mitochondrial carrier oxoglutarate carrier), member 11
Slc25a13	1449481_at	5.089	5.014	0.949	0.5527	solute carrier family 25 (mitochondrial carrier, adenine nucleotide translocator), member 13
Slc25a14	1417154_at	3.762	3.755	0.995	0.9441	solute carrier family 25 (mitochondrial carrier, brain), member 14
Slc25a15	1420966_at	9.073	8.878	0.874	0.2057	solute carrier family 25 (mitochondrial carrier ornithine transporter), member 15

Gene Symbol	Probe set ID	Expression signal 0h	Expression signal 24h	Fold change	P-value	Gene name
Slc25a15	1420967_at	10.513	10.563	1.040	0.6257	solute carrier family 25 (mitochondrial carrier ornithine transporter), member 15
Slc25a17	1424912_at	3.857	4.121	1.200	0.1346	solute carrier family 25 (mitochondrial carrier, peroxisomal membrane protein), member 17
Slc25a17	1450353_at	1.815	1.689	0.916	0.2397	solute carrier family 25 (mitochondrial carrier, peroxisomal membrane protein), member 17
Slc25a19	1424316_at	2.202	2.082	0.920	0.2265	solute carrier family 25 (mitochondrial deoxynucleotide carrier), member 19
Slc25a19	1424317_at	3.118	2.883	0.850	0.1308	solute carrier family 25 (mitochondrial deoxynucleotide carrier), member 19
Slc25a19	1437459_x_at	2.652	2.409	0.845	0.1006	solute carrier family 25 (mitochondrial deoxynucleotide carrier), member 19
Slc25a20	1423108_at	8.971	9.498	1.440	0.0002	solute carrier family 25 (mitochondrial carnitine/acylcarnitine translocase), member 20
Slc25a20	1423109_s_at	7.664	8.186	1.440	0.0019	solute carrier family 25 (mitochondrial carnitine/acylcarnitine translocase), member 20
Slc25a22	1452653_at	8.311	8.415	1.070	0.4250	solute carrier family 25 (mitochondrial carrier, glutamate), member 22
Slc25a23	1419045_at	3.626	3.928	1.230	0.0086	solute "arrier family 25 "mito"hondrial "arrier /// phosphate "arrier, member 23
Slc25a24	1427483_at	6.994	7.260	1.200	0.0305	solute carrier family 25 (mitochondrial carrier, phosphate carrier), member 24
Slc25a25	1424735_at	4.804	4.748	0.962	0.5863	solute carrier family 25 (mitochondrial carrier, phosphate carrier), member 25
Slc25a26	1428929_s_at	3.783	3.714	0.954	0.4403	solute carrier family 25 (mitochondrial carrier, phosphate carrier), member 26
Slc25a26	1453886_a_at	2.068	2.099	1.020	0.7709	solute carrier family 25 (mitochondrial carrier, phosphate carrier), member 26
Slc25a28	1424776_a_at	6.754	7.270	1.430	0.0018	solute carrier family 25, member 28
Slc25a29	1423979_a_at	3.589	3.409	0.883	0.0657	solute carrier family 25 (mitochondrial carrier, palmitoylcarnitine transporter), member 29
Slc25a29	1423980_at	2.457	2.214	0.845	0.0639	solute carrier family 25 (mitochondrial carrier, palmitoylcarnitine transporter), member 29
Slc25a29	1423981_x_at	1.981	1.967	0.990	0.8758	solute carrier family 25 (mitochondrial carrier, palmitoylcarnitine transporter), member 29
Slc25a29	1438187_at	1.835	1.852	1.010	0.8551	solute carrier family 25 (mitochondrial carrier, palmitoylcarnitine transporter), member 29
Slc25a29	1438188_x_at	1.833	1.743	0.940	0.4402	solute carrier family 25 (mitochondrial carrier, palmitoylcarnitine transporter), member 29
Slc25a30	1420835_at	2.752	3.129	1.300	0.0618	solute carrier family 25, member 30
Slc25a30	1420836_at	2.863	3.534	1.590	0.0084	solute carrier family 25, member 30
Slc25a30	1425948_a_at	1.735	1.815	1.060	0.5149	solute carrier family 25, member 30
Slc25a30	1425949_at	1.988	1.970	0.988	0.8380	solute carrier family 25, member 30
Slc25a30	1450018_s_at	3.218	3.449	1.170	0.0364	solute carrier family 25, member 30
Slc25a35	1451489_at	2.930	2.750	0.882	0.1513	solute carrier family 25, member 35
Slc25a36	1419656_at	6.266	7.058	1.730	0.0002	solute carrier family 25, member 36
Slc25a36	1419657_a_at	9.097	9.703	1.520	0.0007	solute carrier family 25, member 36
Slc25a37	1417750_a_at	7.181	7.266	1.060	0.3635	solute carrier family 25, member 37
**Slc26**						
Slc26a1	1451239_a_at	2.037	2.206	1.120	0.0808	solute carrier family 26 (sulfate transporter), member 1
Slc26a2	1421145_at	5.368	5.865	1.410	0.0028	solute carrier family 26 (sulfate transporter), member 2
Slc26a3	1421445_at	8.504	8.737	1.180	0.1933	solute carrier family 26, member 3
Slc26a3	1427547_a_at	8.270	8.569	1.230	0.0204	solute carrier family 26, member 3

Gene Symbol	Probe set ID	Expression signal 0h	Expression signal 24h	Fold change	P-value	Gene name
Slc26a4	1419725_at	1.802	1.761	0.972	0.7329	solute carrier family 26, member 4
Slc26a5	1421725_at	2.378	2.484	1.080	0.2517	solute carrier family 26, member 5
Slc26a6	1416275_at	9.989	10.218	1.170	0.1031	solute carrier family 26, member 6
Slc26a7	1425841_at	1.982	2.004	1.020	0.8632	solute carrier family 26, member 7
Slc26a8	1425915_at	2.857	2.856	0.999	0.9909	solute carrier family 26, member 8
**Slc27**						
Slc27a1	1422811_at	2.629	2.672	1.030	0.6415	solute carrier family 27 (fatty acid transporter), member 1
Slc27a2	1416316_at	10.095	10.774	1.600	0.0003	solute carrier family 27 (fatty acid transporter), member 2
Slc27a3	1427180_at	1.381	1.345	0.975	0.6676	solute carrier family 27 (fatty acid transporter), member 3
Slc27a4	1424441_at	10.724	10.411	0.805	0.0170	solute carrier family 27 (fatty acid transporter), member 4
Slc27a5	1449112_at	2.270	2.188	0.945	0.3655	solute carrier family 27 (fatty acid transporter), member 5
**Slc28**						
Slc28a2 ///	1450639_at	10.296	9.777	0.698	0.0134	solute carrier family 28 (sodium-coupled nucleoside transporter), member 2
Slc28a3	1419570_at	3.347	3.230	0.922	0.2998	solute carrier family 28 (sodium-coupled nucleoside transporter), member 3
Slc28a3	1419571_at	3.449	3.340	0.928	0.2549	solute carrier family 28 (sodium-coupled nucleoside transporter), member 3
**Slc29**						
Slc29a1	1451782_a_at	2.794	2.805	1.010	0.9252	solute carrier family 29 (nucleoside transporters), member 1
Slc29a2	1448257_at	1.549	1.606	1.040	0.6139	solute carrier family 29 (nucleoside transporters), member 2
Slc29a3	1451013_at	2.188	1.988	0.871	0.1614	solute carrier family 29 (nucleoside transporters), member 3
Slc29a3	1455731_at	3.589	3.840	1.190	0.0724	solute carrier family 29 (nucleoside transporters), member 3
Slc29a4	1424900_at	2.232	2.201	0.979	0.7453	solute carrier family 29 (nucleoside transporters), member 4
**Slc30**						
Slc30a1	1422786_at	4.530	4.479	0.965	0.7722	solute carrier family 30 (zinc transporter), member 1
Slc30a2	1427339_at	5.160	6.079	1.890	0.0002	solute carrier family 30 (zinc transporter), member 2
Slc30a3	1460654_at	3.166	3.086	0.946	0.4602	solute carrier family 30 (zinc transporter), member 3
Slc30a4	1418843_at	9.259	9.448	1.140	0.2072	solute carrier family 30 (zinc transporter), member 4
Slc30a5	1422497_at	8.557	8.492	0.956	0.4683	solute carrier family 30 (zinc transporter), member 5
Slc30a6	1424241_at	6.856	6.981	1.090	0.2729	solute carrier family 30 (zinc transporter), member 6
Slc30a7	1450697_at	4.805	4.778	0.982	0.8909	solute carrier family 30 (zinc transporter), member 7
Slc30a9	1426668_at	5.339	5.249	0.940	0.5496	solute carrier family 30 (zinc transporter), member 9
**Slc31**						
Slc31a1	1433750_at	7.636	7.727	1.060	0.3415	solute carrier family 31, member 1
Slc31a1	1455285_at	7.848	7.686	0.894	0.1249	solute carrier family 31, member 1
Slc31a2	1416654_at	3.289	3.249	0.973	0.6757	solute carrier family 31, member 2

Gene Symbol	Probe set ID	Expression signal 0h	Expression signal 24h	Fold change	P-value	Gene name
Slc31a2	1453721_a_at	2.789	2.770	0.988	0.8381	solute carrier family 31, member 2
**Slc32**						
Slc32a1	1456457_at	2.744	2.885	1.100	0.1773	solute carrier family 32 (GABA vesicular transporter), member 1
Slc32a1	1422756_at	1.669	1.709	1.030	0.7797	solute carrier family 32 (GABA vesicular transporter), member 1
**Slc33**						
Slc33a1	1423621_a_at	5.345	5.280	0.955	0.7053	solute carrier family 33 (acetyl-CoA transporter), member 1
**Slc34**						
Slc34a1	1423279_at	1.651	1.814	1.120	0.2528	solute carrier family 34 (sodium phosphate), member 1
Slc34a2	1416854_at	8.784	8.605	0.884	0.6011	solute carrier family 34 (sodium phosphate), member 2
**Slc35**						
Slc35a1	1417538_at	4.636	4.422	0.862	0.2651	solute carrier family 35 (CMP-sialic acid transporter), member 1
Slc35a1	1417539_at	3.599	3.617	1.010	0.9294	solute carrier family 35 (CMP-sialic acid transporter), member 1
Slc35a2	1422827_x_at	4.248	3.927	0.801	0.0376	solute carrier family 35 (UDP-galactose transporter), member 2
Slc35a2	1432533_a_at	4.412	4.275	0.909	0.3076	solute carrier family 35 (UDP-galactose transporter), member 2
Slc35a2	1436664_a_at	4.585	4.336	0.842	0.0725	solute carrier family 35 (UDP-galactose transporter), member 2
Slc35a2	1439433_a_at	6.581	6.365	0.861	0.1797	solute carrier family 35 (UDP-galactose transporter), member 2
Slc35a3	1424579_at	5.896	5.964	1.050	0.6375	solute carrier family 35 (UDP-N-acetylglucosamine (UDP-GlcNAc) transporter), member 3
Slc35a3	1424580_at	5.181	5.198	1.010	0.8918	solute carrier family 35 (UDP-N-acetylglucosamine (UDP-GlcNAc) transporter), member 3
Slc35a4	1416110_at	6.297	6.098	0.871	0.0980	solute carrier family 35, member A4
Slc35a4	1456013_x_at	6.770	6.665	0.930	0.4257	solute carrier family 35, member A4
Slc35a5	1419970_at	2.748	2.807	1.040	0.6650	solute carrier family 35, member A5
Slc35a5	1419971_s_at	7.175	6.815	0.779	0.0986	solute carrier family 35, member A5
Slc35a5	1423457_at	3.726	3.660	0.956	0.4840	solute carrier family 35, member A5
Slc35a5	1423458_at	2.296	2.175	0.920	0.2457	solute carrier family 35, member A5
Slc35b1	1448769_at	9.820	9.865	1.030	0.6642	solute carrier family 35, member B1
Slc35b2	1423927_at	5.682	5.696	1.010	0.9114	solute carrier family 35, member B2
Slc35b3	1448937_at	7.262	7.852	1.510	0.0005	solute carrier family 35, member B3
Slc35b4	1416548_at	2.430	2.347	0.944	0.4464	solute carrier family 35, member B4
Slc35b4	1416549_at	2.702	2.506	0.873	0.1315	solute carrier family 35, member B4
Slc35b4	1416550_at	2.908	2.816	0.938	0.3653	solute carrier family 35, member B4
Slc35c1	1452139_at	7.603	6.958	0.639	0.0052	solute carrier family 35, member C1
Slc35c2	1420054_s_at	7.326	7.583	1.200	0.0948	solute carrier family 35, member C2
Slc35c2	1420055_at	1.495	1.709	1.160	0.0794	solute carrier family 35, member C2
Slc35c2	1460731_at	4.339	4.469	1.090	0.5317	solute carrier family 35, member C2

Gene Symbol	Probe set ID	Expression signal 0h	Expression signal 24h	Fold change	P-value	Gene name
Slc35e4	1434167_at	2.753	2.716	0.974	0.7605	solute carrier family 35, member E4
Slc35e4	1436693_x_at	2.623	2.636	1.010	0.8752	solute carrier family 35, member E4
Slc35f4	1452263_at	2.109	2.070	0.973	0.7214	solute carrier family 35, member F4
Slc35f5	1452059_at	8.284	8.461	1.130	0.1657	solute carrier family 35, member F5
**Slc36**						
**Slc37**						
Slc37a1	1426601_at	6.262	6.434	1.130	0.4453	solute carrier family 37 (glycerol-3-phosphate transporter), member 1
Slc37a2	1452492_a_at	2.721	2.633	0.940	0.3533	solute carrier family 37 (glycerol-3-phosphate transporter), member 2
Slc37a3	1424920_at	3.415	3.476	1.040	0.6457	solute carrier family 37 (glycerol-3-phosphate transporter), member 3
Slc37a3	1453915_a_at	4.589	4.689	1.070	0.4139	solute carrier family 37 (glycerol-3-phosphate transporter), member 3
Slc37a4	1417042_at	7.127	8.317	2.280	0.0010	solute carrier family 37 (glycerol-6-phosphate transporter), member 4
**Slc38**						
Slc38a1	1415903_at	3.150	3.020	0.914	0.3204	solute carrier family 38, member 1
Slc38a2	1426722_at	8.104	8.473	1.290	0.0100	solute carrier family 38, member 2
Slc38a3	1418706_at	2.490	2.477	0.991	0.8918	solute carrier family 38, member 3
Slc38a4	1428111_at	2.330	2.348	1.010	0.8557	solute carrier family 38, member 4
Slc38a4	1448889_at	1.171	1.228	1.040	0.5545	solute carrier family 38, member 4
Slc38a5	1454622_at	1.229	1.438	1.160	0.0399	solute carrier family 38, member 5
**Slc39**						
Slc39a1	1424424_at	7.847	7.699	0.902	0.5323	solute carrier family 39 (zinc transporter), member 1
Slc39a3	1417881_at	3.898	3.874	0.983	0.8474	solute carrier family 39 (zinc transporter), member 3
Slc39a3	1417882_at	2.939	3.031	1.070	0.4517	solute carrier family 39 (zinc transporter), member 3
Slc39a4	1451139_at	9.600	8.367	0.426	0.0003	solute carrier family 39 (zinc transporter), member 4
Slc39a6	1424674_at	5.200	4.974	0.855	0.1283	solute carrier family 39 (metal ion transporter), member 6
Slc39a6	1424675_at	2.527	2.489	0.974	0.7841	solute carrier family 39 (metal ion transporter), member 6
Slc39a7	1416949_s_at	8.689	8.672	0.988	0.8731	solute carrier family 39 (zinc transporter), member 7
Slc39a8	1416832_at	3.854	4.702	1.800	0.0229	solute carrier family 39 (metal ion transporter), member 8
Slc39a8	1448482_at	2.113	2.578	1.380	0.1231	solute carrier family 39 (metal ion transporter), member 8
Slc39a9	1421249_at	1.444	1.464	1.010	0.8612	solute carrier family 39 (zinc transporter), member 9
Slc39a9	1421250_at	2.626	2.535	0.939	0.4517	solute carrier family 39 (zinc transporter), member 9
Slc39a9	1452765_at	7.789	7.798	1.010	0.9472	solute carrier family 39 (zinc transporter), member 9
Slc39a11	1424905_a_at	4.948	4.905	0.971	0.8051	solute carrier family 39 (metal ion transporter), member 11
Slc39a13	1423926_at	2.720	2.711	0.994	0.9146	solute carrier family 39 (metal ion transporter), member 13
Slc39a14	1425649_at	3.923	3.838	0.943	0.6251	solute carrier family 39 (zinc transporter), member 14

Gene Symbol	Probe set ID	Expression signal 0h	Expression signal 24h	Fold change	P-value	Gene name
Slc39a14	1427035_at	7.352	7.724	1.290	0.0628	solute carrier family 39 (zinc transporter), member 14
**Slc40**						
Slc40a1	1417061_at	8.424	7.706	0.608	0.0099	solute carrier family 40 (iron-regulated transporter), member 1
Slc40a1	1448566_at	8.287	7.315	0.510	0.0084	solute carrier family 40 (iron-regulated transporter), member 1
**Slc41**						
Slc41a1	1460565_at	3.743	3.732	0.992	0.8974	solute carrier family 41, member 1
Slc41a2	1452445_at	7.205	7.047	0.897	0.2884	solute carrier family 41, member 2
Slc41a3	1425439_a_at	3.260	3.359	1.070	0.2920	solute carrier family 41, member 3
Slc41a3	1425440_x_at	2.697	2.802	1.080	0.4621	solute carrier family 41, member 3
Slc41a3	1451707_s_at	2.202	2.413	1.160	0.0367	solute carrier family 41, member 3
**Slc43**						
Slc43a3	1422788_at	3.968	4.488	1.430	0.0029	solute carrier family 43, member 3
**Slc44**						
Slc44a1	1423865_at	4.390	4.561	1.130	0.0947	solute carrier family 44, member 1
Slc44a2	1428065_at	3.968	3.918	0.966	0.7477	solute carrier family 44, member 2
Slc44a2	1438559_x_at	5.096	5.217	1.090	0.4107	solute carrier family 44, member 2
Slc44a2	1438860_a_at	3.895	3.799	0.935	0.4120	solute carrier family 44, member 2
Slc44a3	1425109_at	5.371	5.277	0.937	0.5646	solute carrier family 44, member 3
Slc44a4	1416596_at	8.626	8.875	1.190	0.0484	solute carrier family 44, member 4
**Slc45**						
Slc45a2	1437430_at	2.332	2.280	0.964	0.5679	solute carrier family 45, member 2
Slc45a2	1451055_at	1.517	1.558	1.030	0.7098	solute carrier family 45, member 2
Slc45a3	1426663_s_at	1.700	1.479	0.858	0.0708	solute carrier family 45, member 3
Slc45a3	1426664_x_at	3.329	3.137	0.875	0.0591	solute carrier family 45, member 3
