# Supplementary material for: Gene expression of transporters and phase I/II metabolic enzymes in murine small intestine during fasting
Source: BMC Genomics. 2007 Aug 7;8:267. doi: 10.1186/1471-2164-8-267 (PMC1971072; doi:10.1186/1471-2164-8-267)
Supplement: Additional file 2 — Expression of intestinal detoxification enzymes after a 24 hour fasting period. This file contains the expression data, fold changes, and p-values for all detoxification enzymes in fed and 24 hours fasted mice. [file 1471-2164-8-267-S2.pdf]

Gene Symbol	Probe set ID	Expression signal normal fed mice	Expression signal 24h fasted mice	Fold change	P-value	Gene name
**Cytochrome P450 isoforms**						
Cyp1a1	1422217_a_at	2.869	2.539	0.796	0.006	cytochrome P450, family 1, subfamily a, polypeptide 1
Cyp1a2	1450715_at	1.849	1.921	1.050	0.462	cytochrome P450, family 1, subfamily a, polypeptide 2
Cyp1b1	1416612_at	2.121	2.217	1.070	0.383	cytochrome P450, family 1, subfamily b, polypeptide 1
Cyp1b1	1416613_at	2.985	3.098	1.080	0.292	cytochrome P450, family 1, subfamily b, polypeptide 1
Cyp1b1	1432081_at	2.287	2.193	0.937	0.305	cytochrome P450, family 1, subfamily b, polypeptide 1
Cyp2a4 /// Cyp2a5	1422230_s_at	2.756	2.553	0.869	0.117	cytochrome P450, family 2, subfamily a, polypeptide 4; cytochrome P450, family 2, subfamily a, polypeptide 5
Cyp2a12	1418821_at	1.957	2.038	1.060	0.450	cytochrome P450, family 2, subfamily a, polypeptide 12
Cyp2b9	1419590_at	2.199	2.002	0.873	0.105	cytochrome P450, family 2, subfamily b, polypeptide 9
Cyp2b10	1422257_s_at	9.463	8.872	0.664	0.029	cytochrome P450, family 2, subfamily b, polypeptide 10
Cyp2b10	1425645_s_at	9.362	8.646	0.609	0.012	cytochrome P450, family 2, subfamily b, polypeptide 10
Cyp2b10	1451787_at	8.962	8.261	0.615	0.043	cytochrome P450, family 2, subfamily b, polypeptide 10
Cyp2b13	1449479_at	2.563	2.516	0.968	0.662	cytochrome P450, family 2, subfamily b, polypeptide 13
Cyp2b19	1419731_at	1.732	1.833	1.070	0.281	cytochrome P450, family 2, subfamily b, polypeptide 19
Cyp2c29	1417651_at	2.730	3.800	2.100	0.002	cytochrome P450, family 2, subfamily c, polypeptide 29
Cyp2c37 /// Cyp2c50 /// Cyp2c54	1418653_at	1.893	1.796	0.935	0.386	cytochrome P450, family 2. subfamily c, polypeptide 37; cytochrome P450, family 2, subfamily c, polypeptide 50
Cyp2c37 /// Cyp2c50 /// Cyp2c54	1419094_at	2.457	2.506	1.030	0.584	cytochrome P450, family 2. subfamily c, polypeptide 37; cytochrome P450, family 2, subfamily c, polypeptide 50
Cyp2c38	1452501_at	2.354	2.320	0.977	0.702	cytochrome P450, family 2, subfamily c, polypeptide 38
Cyp2c39	1421363_at	1.781	1.665	0.923	0.256	cytochrome P450, family 2, subfamily c, polypeptide 39
Cyp2c44	1424576_s_at	3.615	3.408	0.867	0.317	cytochrome P450, family 2, subfamily c, polypeptide 44
Cyp2c50 /// Cyp2c54	1455457_at	2.298	2.313	1.010	0.875	cytochrome P450, family 2, subfamily c, polypeptide 50; cytochrome P450, family 2, subfamily c, polypeptide 54
Cyp2c55	1419582_at	4.058	2.412	0.320	0.000	cytochrome P450, family 2, subfamily c, polypeptide 55
Cyp2c65	1429994_s_at	10.366	9.976	0.763	0.022	cytochrome P450, family 2, subfamily c, polypeptide 65
Cyp2c70	1424273_at	2.207	2.348	1.100	0.145	cytochrome P450, family 2, subfamily c, polypeptide 70
Cyp2d9	1419349_a_at	3.270	3.395	1.090	0.309	cytochrome P450, family 2, subfamily d, polypeptide 9
Cyp2d10	1418113_at	2.167	2.585	1.340	0.022	cytochrome P450, family 2, subfamily d, polypeptide 10
Cyp2d13	1425365_a_at	2.231	2.214	0.988	0.866	cytochrome P450, family 2, subfamily d, polypeptide 13
Cyp2d13	1431803_at	2.201	2.097	0.930	0.406	cytochrome P450, family 2, subfamily d, polypeptide 13
Cyp2d22	1419039_at	3.943	4.107	1.120	0.154	cytochrome P450, family 2, subfamily d, polypeptide 22
Cyp2d22	1419040_at	4.621	5.133	1.430	0.011	cytochrome P450, family 2, subfamily d, polypeptide 22

Gene Symbol	Probe set ID	Expression signal 0h	Expression signal 24h	Fold change	P-value	Gene name
Cyp2d26	1448683_at	10.368	10.458	1.060	0.340	cytochrome P450, family 2, subfamily d, polypeptide 26
Cyp2e1	1415994_at	2.763	3.010	1.190	0.242	cytochrome P450, family 2, subfamily e, polypeptide 1
Cyp2f2	1448792_a_at	1.315	1.349	1.020	0.693	cytochrome P450, family 2, subfamily f, polypeptide 2
Cyp2g1	1449565_at	1.799	1.818	1.010	0.840	cytochrome P450, family 2, subfamily g, polypeptide 1
Cyp2j5	1417531_at	2.094	1.931	0.893	0.178	cytochrome P450, family 2, subfamily j, polypeptide 5
Cyp2j5	1417532_at	1.771	1.817	1.030	0.688	cytochrome P450, family 2, subfamily j, polypeptide 5
Cyp2j6	1417952_at	7.723	8.945	2.330	0.000	cytochrome P450, family 2, subfamily j, polypeptide 6
Cyp2j9	1424677_at	3.556	4.115	1.470	0.001	cytochrome P450, family 2, subfamily j, polypeptide 9
Cyp2j13	1426102_at	2.208	2.355	1.110	0.155	cytochrome P450, family 2, subfamily j, polypeptide 13
Cyp2s1	1428283_at	8.370	8.439	1.050	0.537	cytochrome P450, family 2, subfamily s, polypeptide 1
Cyp3a11	1416809_at	10.856	11.196	1.270	0.006	cytochrome P450, family 3, subfamily a, polypeptide 11
Cyp3a13	1419523_at	10.839	11.137	1.230	0.063	cytochrome P450, family 3, subfamily a, polypeptide 13
Cyp3a16	1421741_at	2.551	2.661	1.080	0.325	cytochrome P450, family 3, subfamily a, polypeptide 16
Cyp3a25	1424973_at	10.621	10.688	1.050	0.609	cytochrome P450, family 3, subfamily a, polypeptide 25
Cyp3a41	1419704_at	2.824	3.235	1.330	0.026	cytochrome P450, family 3, subfamily a, polypeptide 41
Cyp3a44	1426064_at	3.972	4.214	1.180	0.039	cytochrome P450, family 3, subfamily a, polypeptide 44
Cyp4a10	1424853_s_at	6.508	8.348	3.580	0.002	cytochrome P450, family 4, subfamily a, polypeptide 10
Cyp4a14	1423257_at	2.009	1.894	0.924	0.314	cytochrome P450, family 4, subfamily a, polypeptide 14
Cyp4b1	1416194_at	9.096	10.230	2.200	0.000	cytochrome P450, family 4, subfamily b, polypeptide 1
Cyp4f13	1418767_at	4.659	4.897	1.180	0.059	cytochrome P450, family 4, subfamily f, polypeptide 13
Cyp4f14	1419559_at	10.747	10.676	0.952	0.446	cytochrome P450, family 4, subfamily f, polypeptide 14
Cyp4f15	1449316_at	1.992	1.992	1.000	0.996	cytochrome P450, family 4, subfamily f, polypeptide 15
Cyp4f16	1417277_at	8.669	8.591	0.947	0.650	cytochrome P450, family 4, subfamily f, polypeptide 16
Cyp4f16	1430173_x_at	4.328	4.245	0.944	0.466	cytochrome P450, family 4, subfamily f, polypeptide 16
Cyp4f16	1430172_a_at	9.552	9.397	0.899	0.327	cytochrome P450, family 4, subfamily f, polypeptide 16
Cyp4f18	1419219_at	2.990	3.101	1.080	0.408	cytochrome P450, family 4, subfamily f, polypeptide 18
Cyp4v3	1417070_at	9.900	10.134	1.180	0.079	cytochrome P450, family 4, subfamily v, polypeptide 3
Cyp4v3	1417071_s_at	10.750	10.943	1.140	0.077	cytochrome P450, family 4, subfamily v, polypeptide 3
Cyp7a1	1422100_at	2.088	1.960	0.915	0.360	cytochrome P450, family 7, subfamily a, polypeptide 1
Cyp7a1	1438743_at	2.622	2.578	0.970	0.726	cytochrome P450, family 7, subfamily a, polypeptide 1
Cyp7b1	1421074_at	1.906	2.132	1.170	0.086	cytochrome P450, family 7, subfamily b, polypeptide 1
Cyp7b1	1421075_s_at	2.848	2.915	1.050	0.459	cytochrome P450, family 7, subfamily b, polypeptide 1
Cyp8b1	1449309_at	1.555	1.639	1.060	0.378	cytochrome P450, family 8, subfamily b, polypeptide 1
Cyp11a1	1448804_at	2.090	2.093	1.000	0.970	cytochrome P450, family 11, subfamily a, polypeptide 1
Cyp11b2	1450574_at	1.393	1.409	1.010	0.923	cytochrome P450, family 11, subfamily b, polypeptide 2
Cyp17a1	1417017_at	1.936	1.832	0.930	0.297	cytochrome P450, family 17, subfamily a, polypeptide 1

Gene Symbol	Probe set ID	Expression signal 0h	Expression signal 24h	Fold change	P-value	Gene name
Cyp19a1	1449920_at	1.609	1.649	1.030	0.638	cytochrome P450, family 19, subfamily a, polypeptide 1
Cyp21a1	1422333_at	2.116	2.070	0.968	0.623	cytochrome P450, family 21, subfamily a, polypeptide 1
Cyp21a1	1455691_at	1.827	1.777	0.966	0.591	cytochrome P450, family 21, subfamily a, polypeptide 1
Cyp24a1	1418866_at	1.970	1.930	0.973	0.670	cytochrome P450, family 24, subfamily a, polypeptide 1
Cyp24a1	1418867_at	2.580	2.564	0.989	0.856	cytochrome P450, family 24, subfamily a, polypeptide 1
Cyp26a1	1419430_at	2.233	2.298	1.050	0.543	cytochrome P450, family 26, subfamily a, polypeptide 1
Cyp27a1	1417590_at	7.600	9.232	3.100	0.000	cytochrome P450, family 27, subfamily a, polypeptide 1
Cyp27b1	1427372_at	1.786	1.670	0.923	0.259	cytochrome P450, family 27, subfamily b, polypeptide 1
Cyp39a1	1418780_at	2.940	3.192	1.190	0.062	cytochrome P450, family 39, subfamily a, polypeptide 1
Cyp46a1	1417709_at	2.425	2.341	0.944	0.429	cytochrome P450, family 46, subfamily a, polypeptide 1
Cyp51	1422533_at	3.831	3.802	0.980	0.847	cytochrome P450, family 51
Cyp51	1422534_at	1.800	1.827	1.020	0.747	cytochrome P450, family 51
Cyp51	1450646_at	6.818	6.501	0.803	0.022	cytochrome P450, family 51
**aldo-keto reductases**						
Akr1a4	1430123_a_at	12.436	12.376	0.959	0.483	aldo-keto reductase family 1, member A4 (aldehyde reductase)
Akr1a4	1430124_x_at	11.109	11.079	0.980	0.750	aldo-keto reductase family 1, member A4 (aldehyde reductase)
Akr1a4	1435011_x_at	11.878	11.809	0.953	0.421	aldo-keto reductase family 1, member A4 (aldehyde reductase)
Akr1a4	1451035_a_at	12.006	11.965	0.972	0.625	aldo-keto reductase family 1, member A4 (aldehyde reductase)
Akr1b3	1437133_x_at	6.709	6.816	1.080	0.297	aldo-keto reductase family 1, member B3 (aldose reductase)
Akr1b3	1456590_x_at	4.613	4.557	0.962	0.792	aldo-keto reductase family 1, member B3 (aldose reductase)
Akr1b7	1423556_at	9.667	10.998	2.520	0.001	aldo-keto reductase family 1, member B7
Akr1b8	1448894_at	5.488	6.026	1.450	0.142	aldo-keto reductase family 1, member B8
Akr1c12	1422000_at	8.632	8.531	0.932	0.494	aldo-keto reductase family 1, member C12
Akr1c12	1450455_s_at	10.544	10.365	0.883	0.333	aldo-keto reductase family 1, member C12
Akr1c13	1418672_at	9.458	9.319	0.908	0.451	aldo-keto reductase family 1, member C13
Akr1c18	1419136_at	3.190	3.142	0.967	0.635	aldo-keto reductase family 1, member C18
Akr1c20	1422061_at	2.476	2.384	0.938	0.432	aldo-keto reductase family 1, member C20
Akr1c20	1425387_at	3.298	3.203	0.936	0.409	aldo-keto reductase family 1, member C20
Akr1c21	1451030_at	2.943	2.954	1.010	0.910	aldo-keto reductase family 1, member C21
Akr1c6	1417085_at	2.362	2.257	0.929	0.318	aldo-keto reductase family 1, member C6
Akr1d1	1425771_at	1.822	1.733	0.940	0.377	aldo-keto reductase family 1, member D1
Akr1d1	1455100_at	3.141	3.150	1.010	0.927	aldo-keto reductase family 1, member D1
Akr1e1	1417826_at	6.836	6.753	0.944	0.524	aldo-keto reductase family 1, member E1
Akr7a5	1417294_at	8.858	9.118	1.200	0.034	aldo-keto reductase family 7, member A5 (aflatoxin aldehyde reductase)
Akr7a5	1438315_x_at	9.570	9.675	1.080	0.298	aldo-keto reductase family 7, member A5 (aflatoxin aldehyde reductase)

Gene Symbol	Probe set ID	Expression signal 0h	Expression signal 24h	Fold change	P-value	Gene name
Miox	1416460_at	1.418	1.515	1.070	0.301	myo-inositol oxygenase
**epoxide hydrolases**						
Ephx1	1422438_at	6.359	5.688	0.628	0.045	epoxide hydrolase 1, microsomal
Ephx2	1448499_a_at	11.220	11.324	1.080	0.429	epoxide hydrolase 2, cytoplasmic
**glucuronosyl transferases**						
Ugt1a2 /// Ugt1a6a /// Ugt1a10 /// Ugt1a7c /// Ugt1a5 /// Ugt1a9 /// Ugt1a6b /// Ugt1a1	1424783_a_at	10.699	10.712	1.010	0.884	UDP glucuronosyltransferase 1 family, polypeptide A2; UDP glucuronosyltransferase 1 family, polypeptide A6A; UDP glycosyltransferase 1 family, polypeptide A10; UDP glucuronosyltransferase 1 family, polypeptide A7C; UDP glucuronosyltransferase 1 family, polypeptide A5; UDP glucuronosyltransferase 1 family, polypeptide A9; UDP glucuronosyltransferase 1 family, polypeptide A6B; UDP glucuronosyltransferase 1 family, polypeptide A1
Ugt1a2 /// Ugt1a6a /// Ugt1a10 /// Ugt1a7c /// Ugt1a5 /// Ugt1a9 /// Ugt1a6b /// Ugt1a1	1426260_a_at	11.719	11.748	1.020	0.737	UDP glucuronosyltransferase 1 family, polypeptide A2; UDP glucuronosyltransferase 1 family, polypeptide A6A; UDP glycosyltransferase 1 family, polypeptide A10; UDP glucuronosyltransferase 1 family, polypeptide A7C; UDP glucuronosyltransferase 1 family, polypeptide A5; UDP glucuronosyltransferase 1 family, polypeptide A9; UDP glucuronosyltransferase 1 family, polypeptide A6B; UDP glucuronosyltransferase 1 family, polypeptide A1
Ugt1a2 /// Ugt1a6a /// Ugt1a10 /// Ugt1a7c /// Ugt1a5 /// Ugt1a9 /// Ugt1a6b /// Ugt1a1	1426261_s_at	10.691	10.820	1.090	0.181	UDP glucuronosyltransferase 1 family, polypeptide A2; UDP glucuronosyltransferase 1 family, polypeptide A6A; UDP glycosyltransferase 1 family, polypeptide A10; UDP glucuronosyltransferase 1 family, polypeptide A7C; UDP glucuronosyltransferase 1 family, polypeptide A5; UDP glucuronosyltransferase 1 family, polypeptide A9; UDP glucuronosyltransferase 1 family, polypeptide A6B; UDP glucuronosyltransferase 1 family, polypeptide A1
Ugt2a1 /// Ugt2a2	1421484_at	2.454	2.465	1.010	0.928	UDP glucuronosyltransferase 2 family, polypeptide A1; UDP glucuronosyltransferase 2 family, polypeptide A2
Ugt2a3	1450133_at	8.467	8.349	0.922	0.337	UDP glucuronosyltransferase 2 family, polypeptide A3
Ugt2b1	1424934_at	2.073	1.985	0.941	0.477	UDP glucuronosyltransferase 2 family, polypeptide B1
Ugt2b34	1427960_at	10.564	10.335	0.853	0.066	UDP glucuronosyltransferase 2 family, polypeptide B34
Ugt2b34	1427961_s_at	10.887	10.642	0.844	0.055	UDP glucuronosyltransferase 2 family, polypeptide B34
Ugt2b37	1449890_at	2.504	2.618	1.080	0.437	UDP glucuronosyltransferase 2 family, polypeptide B37
Ugt2b38	1423397_at	1.623	1.707	1.060	0.531	UDP glucuronosyltransferase 2 family, polypeptide B5
Ugt2b5	1419622_at	8.868	8.111	0.591	0.023	UDP glucuronosyltransferase 2 family, polypeptide B5
Ugt3a1	1424493_s_at	2.185	2.142	0.971	0.649	UDP glycosyltransferases 3 family, polypeptide A1
Ugt3a1	1451373_at	1.924	1.892	0.978	0.726	UDP glycosyltransferases 3 family, polypeptide A1

Gene Symbol	Probe set ID	Expression signal 0h	Expression signal 24h	Fold change	P-value	Gene name
Ugt3a2	1423968_at	2.270	2.282	1.010	0.897	UDP glycosyltransferases 3 family, polypeptide A2
Ugt8a	1419063_at	1.895	2.179	1.220	0.055	UDP galactosyltransferase 8A
Ugt8a	1419064_a_at	1.904	1.839	0.955	0.511	UDP galactosyltransferase 8A
**glutathione reductases**						
Gsr	1421816_at	8.496	7.998	0.708	0.052	glutathione reductase 1
Gsr	1421817_at	7.141	6.697	0.735	0.021	glutathione reductase 1
Gss	1448273_at	6.102	6.002	0.933	0.355	glutathione synthetase
**glutathione peroxidases**						
Gpx1	1460671_at	10.579	10.545	0.977	0.729	glutathione peroxidase 1
Gpx2	1449279_at	11.307	10.470	0.560	0.011	glutathione peroxidase 2
Gpx3	1449106_at	6.865	7.369	1.420	0.061	glutathione peroxidase 3
Gpx4	1451695_a_at	10.542	10.413	0.914	0.240	glutathione peroxidase 4
Gpx5	1420698_at	2.224	2.250	1.020	0.770	glutathione peroxidase 5
Gpx6	1452135_at	1.863	1.701	0.894	0.243	glutathione peroxidase 6
Gpx7	1417836_at	2.254	2.454	1.150	0.076	glutathione peroxidase 7
**glutathione transferases**						
Gsta1 /// Gsta2	1421041_s_at	11.813	11.500	0.805	0.198	glutathione S-transferase, alpha 1 (Ya); glutathione S-transferase, alpha 2 (Yc2)
Gsta2	1421040_a_at	5.115	4.090	0.492	0.036	glutathione S-transferase, alpha 2 (Yc2)
Gsta3	1423436_at	5.346	4.561	0.580	0.041	glutathione S-transferase, alpha 3
Gsta3	1423437_at	3.100	2.732	0.775	0.040	glutathione S-transferase, alpha 3
Gsta4	1416368_at	9.166	8.741	0.745	0.086	glutathione S-transferase, alpha 4
Gstk1	1452823_at	9.035	8.620	0.750	0.003	glutathione S-transferase kappa 1
Gstm1	1416416_x_at	10.234	9.158	0.474	0.022	glutathione S-transferase, mu 1
Gstm1	1448330_at	9.793	8.488	0.405	0.017	glutathione S-transferase, mu 1
Gstm1	1425626_at	2.587	2.174	0.751	0.012	glutathione S-transferase, mu 1
Gstm1	1425627_x_at	3.948	3.480	0.723	0.049	glutathione S-transferase, mu 1
Gstm2	1416411_at	6.321	5.882	0.738	0.027	glutathione S-transferase, mu 2
Gstm3	1427473_at	6.122	4.743	0.384	0.001	glutathione S-transferase, mu 3
Gstm3	1427474_s_at	9.948	8.475	0.360	0.003	glutathione S-transferase, mu 3
Gstm4	1424835_at	5.239	3.784	0.365	0.001	glutathione S-transferase, mu 4
Gstm5	1416842_at	7.417	6.809	0.656	0.000	glutathione S-transferase, mu 5
Gstm6	1422072_a_at	5.814	4.952	0.550	0.020	glutathione S-transferase, mu 6
Gstm7	1419072_at	4.076	3.589	0.713	0.020	glutathione S-transferase, mu 7

Gene Symbol	Probe set ID	Expression signal 0h	Expression signal 24h	Fold change	P-value	Gene name
Gstm7	1425946_at	2.429	2.343	0.942	0.385	glutathione S-transferase, mu 7
Gsto1	1416531_at	10.495	10.458	0.974	0.734	glutathione S-transferase omega 1
Gsto1	1456036_x_at	10.228	10.303	1.050	0.732	glutathione S-transferase omega 1
Gstp1	1449575_a_at	12.098	11.631	0.724	0.003	glutathione S-transferase, pi 1
Gstt1	1418186_at	7.451	6.657	0.577	0.000	glutathione S-transferase, theta 1
Gstt2	1417883_at	7.189	7.401	1.160	0.148	glutathione S-transferase, theta 2
Gstt3	1423891_at	5.490	5.217	0.828	0.084	glutathione S-transferase, theta 3
Gstz1	1427552_a_at	5.337	5.782	1.360	0.011	glutathione transferase zeta 1 (maleylacetoacetate isomerase)
Mgst1	1415897_a_at	10.915	11.323	1.330	0.001	microsomal glutathione S-transferase 1
Mgst1	1415898_at	1.852	1.886	1.020	0.717	microsomal glutathione S-transferase 1
Mgst2	1452592_at	10.352	10.010	0.789	0.005	microsomal glutathione S-transferase 2
Mgst3	1448300_at	11.183	11.284	1.070	0.409	microsomal glutathione S-transferase 3
Mgst3	1450563_at	3.075	3.002	0.951	0.515	microsomal glutathione S-transferase 3
**N-acetyltransferases**						
Nat1	1421758_at	1.739	1.738	1.000	0.993	N-acetyltransferase 1 (arylamine N-acetyltransferase)
Nat3	1422961_at	1.904	1.765	1.100	0.215	N-acetyltransferase 3
Nat2	1449981_a_at	7.303	7.091	1.160	0.129	N-acetyltransferase 2 (arylamine N-acetyltransferase)
Gcnt1	1449538_a_at	2.950	2.941	0.993	0.918	glucosaminyl (N-acetyl) transferase 1, core 2
**sulfotransferases**						
Chst1	1449147_at	3.019	2.917	0.932	0.240	carbohydrate (keratan sulfate Gal-6) sulfotransferase 1
Chst10	1426620_at	2.088	1.985	0.931	0.273	carbohydrate sulfotransferase 10
Chst11	1450509_at	2.480	2.665	1.140	0.215	carbohydrate sulfotransferase 11
Chst11 /// Phactr1	1456606_a_at	3.522	3.575	1.040	0.647	carbohydrate sulfotransferase 11; phosphatase and actin regulator 1
Chst12	1448477_at	3.270	3.327	1.040	0.651	carbohydrate sulfotransferase 12
Chst2	1422758_at	2.546	2.405	0.907	0.222	carbohydrate sulfotransferase 2
Chst3	1460322_at	2.590	2.548	0.972	0.698	carbohydrate (chondroitin 6/keratan) sulfotransferase 3
Chst4	1453393_a_at	4.458	4.904	1.360	0.001	carbohydrate (chondroitin 6/keratan) sulfotransferase 4
Chst5	1419320_at	2.785	2.737	0.968	0.647	carbohydrate (N-acetylglucosamine 6-O) sulfotransferase 5
Chst7	1449402_at	2.335	2.308	0.982	0.791	carbohydrate (N-acetylglucosamino) sulfotransferase 7
D4st1	1426866_at	2.396	2.335	0.958	0.530	dermatan 4 sulfotransferase 1
Gal3st1	1454078_a_at	7.425	7.768	1.270	0.043	galactose-3-O-sulfotransferase 1
Hs2st1	1422739_at	1.994	1.952	0.972	0.641	heparan sulfate 2-O-sulfotransferase 1
Hs2st1	1450729_at	3.497	3.346	0.901	0.419	heparan sulfate 2-O-sulfotransferase 1
Hs2st1	1450730_at	3.335	3.351	1.010	0.883	heparan sulfate 2-O-sulfotransferase 1

Gene Symbol	Probe set ID	Expression signal 0h	Expression signal 24h	Fold change	P-value	Gene name
Hs3st1	1423450_a_at	2.327	2.173	0.899	0.111	heparan sulfate (glucosamine) 3-O-sulfotransferase 1
Hs3st3a1	1435622_at	2.984	2.879	0.930	0.462	heparan sulfate (glucosamine) 3-O-sulfotransferase 3A1
Hs3st3b1	1421331_at	2.307	2.255	0.965	0.659	heparan sulfate (glucosamine) 3-O-sulfotransferase 3B1
Hs3st3b1	1421332_at	2.855	2.835	0.986	0.853	heparan sulfate (glucosamine) 3-O-sulfotransferase 3B1
Hs6st1	1417293_at	5.056	5.060	1.000	0.988	heparan sulfate 6-O-sulfotransferase 1
Hs6st2	1420938_at	1.259	1.340	1.060	0.373	heparan sulfate 6-O-sulfotransferase 2
Hs6st2	1420939_at	1.837	1.856	1.010	0.864	heparan sulfate 6-O-sulfotransferase 2
Hs6st2	1450047_at	2.267	2.305	1.030	0.734	heparan sulfate 6-O-sulfotransferase 2
Hs6st3	1421599_at	2.312	2.420	1.080	0.267	heparan sulfate 6-O-sulfotransferase 3
Ndst1	1422044_at	3.791	3.868	1.060	0.653	N-deacetylase/N-sulfotransferase (heparan glucosaminyl) 1
Ndst1	1428367_at	6.703	6.875	1.130	0.197	N-deacetylase/N-sulfotransferase (heparan glucosaminyl) 1
Ndst1	1460436_at	8.606	8.644	1.030	0.727	N-deacetylase/N-sulfotransferase (heparan glucosaminyl) 1
Ndst2	1417931_at	3.124	3.319	1.150	0.082	N-deacetylase/N-sulfotransferase (heparan glucosaminyl) 2
Ndst3	1453777_a_at	1.795	1.887	1.070	0.342	N-deacetylase/N-sulfotransferase (heparan glucosaminyl) 3
Ndst4	1421318_at	1.848	1.661	0.878	0.144	N-deacetylase/N-sulfotransferase (heparin glucosaminyl) 4
Ndst4	1450195_at	1.935	1.762	0.887	0.188	N-deacetylase/N-sulfotransferase (heparin glucosaminyl) 4
Sult1a1	1427345_a_at	3.719	4.638	1.890	0.009	sulfotransferase family 1A, phenol-preferring, member 1
Sult1b1	1418940_at	10.068	10.050	0.987	0.843	sulfotransferase family 1B, member 1
Sult1c1	1420470_at	2.371	2.272	0.933	0.273	sulfotransferase family, cytosolic, 1C, member 1
Sult1c2	1449409_at	2.792	2.785	0.995	0.957	sulfotransferase family, cytosolic, 1C, member 2
Sult1d1	1418138_at	9.558	10.298	1.670	0.002	sulfotransferase family 1D, member 1
Sult1d1	1448973_at	9.778	10.260	1.400	0.021	sulfotransferase family 1D, member 1
Sult1e1	1420447_at	2.776	2.668	0.927	0.317	sulfotransferase family 1E, member 1
Sult2a2	1419528_at	2.001	2.036	1.020	0.721	sulfotransferase family 2A, dehydroepiandrosterone (DHEA)-preferring, member 2
Sult2b1	1417335_at	9.220	9.037	0.881	0.130	sulfotransferase family, cytosolic, 2B, member 1
Sult3a1	1421669_at	2.124	2.018	0.929	0.283	sulfotransferase family 3A, member 1
Sult4a1	1421606_a_at	2.322	2.246	0.949	0.477	sulfotransferase family 4A, member 1
Sult5a1	1449816_at	2.479	2.572	1.070	0.483	sulfotransferase family 5A, member 1
