# Supplementary material for: Gene expression of transporters and phase I/II metabolic enzymes in murine small intestine during fasting
Source: BMC Genomics. 2007 Aug 7;8:267. doi: 10.1186/1471-2164-8-267 (PMC1971072; doi:10.1186/1471-2164-8-267)
Supplement: Additional file 3 — Expression of intestinal ABC transporters after a 24 hour fasting period. This file contains the expression data, fold changes, and p-values for all ABC transporters in fed and 24 hours fasted mice. [file 1471-2164-8-267-S3.pdf]

Gene Symbol	Probe set ID	Expression signal normal fed mice	Expression signal 24h fasted mice	Fold change	P-value	Gene name
Abca1	1421839_at	3.030	3.346	1.240	0.008	ATP-binding cassette, sub-family A (ABC1), member 1
Abca1	1421840_at	5.659	6.872	2.320	0.000	ATP-binding cassette, sub-family A (ABC1), member 1
Abca1	1450392_at	2.871	3.134	1.200	0.033	ATP-binding cassette, sub-family A (ABC1), member 1
Abca2	1449302_at	2.592	2.495	0.935	0.367	ATP-binding cassette, sub-family A (ABC1), member 2
Abca3	1425496_at	4.815	4.767	0.967	0.759	ATP-binding cassette, sub-family A (ABC1), member 3
Abca3	1451731_at	6.648	6.768	1.090	0.392	ATP-binding cassette, sub-family A (ABC1), member 3
Abca3	1451869_at	2.250	2.479	1.170	0.048	ATP-binding cassette, sub-family A (ABC1), member 3
Abca4	1449588_at	1.976	1.949	0.982	0.819	ATP-binding cassette, sub-family A (ABC1), member 4
Abca7	1419238_at	5.055	4.865	0.877	0.166	ATP-binding cassette, sub-family A (ABC1), member 7
Abca8a	1427371_at	3.249	3.683	1.350	0.048	ATP-binding cassette, sub-family A (ABC1), member 8a
Abca8b	1427546_at	2.363	2.432	1.050	0.440	ATP-binding cassette, sub-family A (ABC1), member 8b
Abcb1a	1419758_at	8.166	8.243	1.050	0.737	ATP-binding cassette, sub-family B (MDR/TAP), member 1A
Abcb1a	1419759_at	9.139	9.104	0.976	0.886	ATP-binding cassette, sub-family B (MDR/TAP), member 1A
Abcb1b	1418872_at	2.632	2.773	1.100	0.265	ATP-binding cassette, sub-family B (MDR/TAP), member 1B
Abcb1b	1449594_at	2.950	2.968	1.010	0.907	ATP-binding cassette, sub-family B (MDR/TAP), member 1B
Tap1	1416016_at	7.273	7.076	0.872	0.390	transporter 1, ATP-binding cassette, sub-family B (MDR/TAP)
Tap1	1448177_at	2.368	2.213	0.898	0.168	transporter 1, ATP-binding cassette, sub-family B (MDR/TAP)
Tap2	1453913_a_at	5.644	5.307	0.792	0.052	transporter 2, ATP-binding cassette, sub-family B (MDR/TAP)
Abcb4	1449818_at	2.744	2.726	0.988	0.894	ATP-binding cassette, sub-family B (MDR/TAP), member 4
Abcb6	1422524_at	5.231	5.138	0.938	0.490	ATP-binding cassette, sub-family B (MDR/TAP), member 6
Abcb7	1419931_at	2.973	2.780	0.874	0.160	ATP-binding cassette, sub-family B (MDR/TAP), member 7
Abcb7	1427490_at	4.014	4.013	1.000	0.998	ATP-binding cassette, sub-family B (MDR/TAP), member 7
Abcb7	1435006_s_at	6.883	6.601	0.822	0.012	ATP-binding cassette, sub-family B (MDR/TAP), member 7
Abcb8	1422015_a_at	4.736	4.438	0.813	0.044	ATP-binding cassette, sub-family B (MDR/TAP), member 8
Abcb8	1423713_at	4.407	4.166	0.846	0.048	ATP-binding cassette, sub-family B (MDR/TAP), member 8
Abcb9	1416263_at	3.259	3.171	0.941	0.401	ATP-binding cassette, sub-family B (MDR/TAP), member 9
Abcb9	1416264_at	2.812	2.729	0.944	0.421	ATP-binding cassette, sub-family B (MDR/TAP), member 9
Abcb9	1430394_a_at	2.709	2.760	1.040	0.684	ATP-binding cassette, sub-family B (MDR/TAP), member 9
Abcb10	1416402_at	3.798	3.798	1.000	0.999	ATP-binding cassette, sub-family B (MDR/TAP), member 10
Abcb10	1416403_at	2.903	2.818	0.943	0.386	ATP-binding cassette, sub-family B (MDR/TAP), member 10
Abcb10	1454265_a_at	1.663	1.557	0.929	0.239	ATP-binding cassette, sub-family B (MDR/TAP), member 10
Abcb11	1449817_at	1.539	1.537	0.999	0.986	ATP-binding cassette, sub-family B (MDR/TAP), member 11
Abcc1	1421378_s_at	2.294	2.116	0.884	0.135	ATP-binding cassette, sub-family C (CFTR/MRP), member 1
Abcc1	1452233_at	3.652	3.488	0.892	0.179	ATP-binding cassette, sub-family C (CFTR/MRP), member 1
Abcc2	1450109_s_at	4.913	4.560	0.783	0.126	ATP-binding cassette, sub-family C (CFTR/MRP), member 2
Abcc3	1428988_at	8.074	7.361	0.610	0.024	ATP-binding cassette, sub-family C (CFTR/MRP), member 3
Abcc5	1418042_a_at	2.999	3.022	1.020	0.865	ATP-binding cassette, sub-family C (CFTR/MRP), member 5
Abcc5	1418043_at	1.735	1.810	1.050	0.430	ATP-binding cassette, sub-family C (CFTR/MRP), member 5

Gene Symbol	Probe set ID	Expression signal 0h	Expression signal 24h	Fold change	P-value	Gene name
Abcc5	1427565_a_at	2.792	2.758	0.977	0.780	ATP-binding cassette, sub-family C (CFTR/MRP), member 5
Abcc5	1435683_a_at	4.286	4.489	1.150	0.190	ATP-binding cassette, sub-family C (CFTR/MRP), member 5
Abcc5	1435684_at	2.849	2.875	1.020	0.763	ATP-binding cassette, sub-family C (CFTR/MRP), member 5
Abcc5	1435685_x_at	2.693	2.697	1.000	0.965	ATP-binding cassette, sub-family C (CFTR/MRP), member 5
Abcc5	1438056_x_at	2.546	2.620	1.050	0.517	ATP-binding cassette, sub-family C (CFTR/MRP), member 5
Abcc6	1421212_at	2.143	2.151	1.010	0.929	ATP-binding cassette, sub-family C (CFTR/MRP), member 6
Abcc8	1455765_a_at	3.388	3.495	1.080	0.596	ATP-binding cassette, sub-family C (CFTR/MRP), member 8
Abcc9	1420408_a_at	2.498	2.696	1.150	0.093	ATP-binding cassette, sub-family C (CFTR/MRP), member 9
Abcc10	1428009_a_at	3.314	3.091	0.857	0.341	ATP-binding cassette, sub-family C (CFTR/MRP), member 10
Abcd1	1418838_at	8.392	8.174	0.860	0.125	ATP-binding cassette, sub-family D (ALD), member 1
Abcd2	1419748_at	1.985	2.088	1.070	0.339	ATP-binding cassette, sub-family D (ALD), member 2
Abcd3	1416679_at	10.760	10.665	0.936	0.340	ATP-binding cassette, sub-family D (ALD), member 3
Abcd4	1419572_a_at	4.353	4.369	1.010	0.883	ATP-binding cassette, sub-family D (ALD), member 4
Abcd4	1427829_at	1.513	1.511	0.999	0.983	ATP-binding cassette, sub-family D (ALD), member 4
Abce1	1416014_at	3.902	3.683	0.859	0.174	ATP-binding cassette, sub-family E (OABP), member 1
Abce1	1416015_s_at	5.621	5.736	1.080	0.378	ATP-binding cassette, sub-family E (OABP), member 1
Abcf1	1420157_s_at	7.112	7.138	1.020	0.824	ATP-binding cassette, sub-family F (GCN20), member 1
Abcf1	1420158_s_at	2.789	2.608	0.882	0.091	ATP-binding cassette, sub-family F (GCN20), member 1
Abcf1	1426921_at	3.819	3.872	1.040	0.561	ATP-binding cassette, sub-family F (GCN20), member 1
Abcf1	1427444_at	1.734	1.797	1.040	0.477	ATP-binding cassette, sub-family F (GCN20), member 1
Abcf1	1452236_at	4.391	4.506	1.080	0.431	ATP-binding cassette, sub-family F (GCN20), member 1
Abcf1	1452429_s_at	4.753	4.585	0.890	0.112	ATP-binding cassette, sub-family F (GCN20), member 1
Abcf2	1423863_at	3.896	3.811	0.943	0.426	ATP-binding cassette, sub-family F (GCN20), member 2
Abcf3	1426747_at	4.801	4.819	1.010	0.893	ATP-binding cassette, sub-family F (GCN20), member 3
Abcf3	1426748_s_at	2.717	2.672	0.969	0.615	ATP-binding cassette, sub-family F (GCN20), member 3
Abcf3	1452177_at	2.432	2.431	0.999	0.987	ATP-binding cassette, sub-family F (GCN20), member 3
Abcg1	1423570_at	3.712	3.712	1.000	0.999	ATP-binding cassette, sub-family G (WHITE), member 1
Abcg2	1422906_at	9.955	10.054	1.070	0.310	ATP-binding cassette, sub-family G (WHITE), member 2
Abcg3	1421168_at	2.832	2.646	0.879	0.150	ATP-binding cassette, sub-family G (WHITE), member 3
Abcg3	1450141_at	2.485	2.557	1.050	0.584	ATP-binding cassette, sub-family G (WHITE), member 3
Abcg4	1424437_s_at	1.341	1.248	0.937	0.336	ATP-binding cassette, sub-family G (WHITE), member 4
Abcg4	1427732_s_at	2.123	2.203	1.060	0.370	ATP-binding cassette, sub-family G (WHITE), member 4
Abcg5	1419393_at	10.474	10.853	1.300	0.014	ATP-binding cassette, sub-family G (WHITE), member 5
Abcg8	1420656_at	7.611	8.463	1.810	0.000	ATP-binding cassette, sub-family G (WHITE), member 8
